# SIRPα antibody combined with oncolytic virus OH2 protects against tumours by activating innate immunity and reprogramming the tumour immune microenvironment

**DOI:** 10.1186/s12916-022-02574-z

**Published:** 2022-10-31

**Authors:** Defeng Kong, Zhenrong Yang, Guoliang Li, Quanyou Wu, Zhaoru Gu, Duo Wan, Qi Zhang, Xiaoli Zhang, Shujun Cheng, Binlei Liu, Kaitai Zhang, Wen Zhang

**Affiliations:** 1 State Key Laboratory of Molecular Oncology, Department of Etiology and Carcinogenesis, National Cancer Center/National Clinical Research Center for Cancer/Cancer Hospital, Chinese Academy of Medical Sciences and Peking Union Medical College, Beijing, 100021 China; 2 National “111” Center for Cellular Regulation and Molecular Pharmaceutics, Key Laboratory of Fermentation Engineering (Ministry of Education), Hubei Provincial Cooperative Innovation Center of Industrial Fermentation, College of Bioengineering, Hubei University of Technology, Wuhan, 430068 China; 3 Department of Immunology, National Cancer Center/National Clinical Research Center for Cancer/Cancer Hospital, Chinese Academy of Medical Sciences and Peking Union Medical College, Beijing, 100021 China

**Keywords:** Oncolytic virus, Oncolytic herpes simplex virus 2, SIRPα, Macrophage, Tumour microenvironment environment

## Abstract

**Background:**

The combination of oncolytic viruses (OVs) with immune checkpoint blockades is a research hotspot and has shown good efficacy. Here, we present the first attempt to combine oncolytic herpes simplex virus 2 (OH2) with an anti-SIRPα antibody as an antitumour treatment. Our results provide unique insight into the combination of innate immunity with OV.

**Methods:**

We verified the polarization and activation of OH2 in RAW264.7 cells in vitro. Subsequently, we evaluated the antitumour ability of OH2 and anti-SIRPα combined therapy in a tumour-bearing mouse model. RNA-seq and Single-cell RNA-seq were used to characterize the changes in the tumour microenvironment.

**Results:**

The OH2 lysates effectively stimulated RAW264.7 cells to polarize towards the M1 but not the M2 phenotype and activated the function of the M1 phenotype in vitro. In the macrophage clearance experiment, OH2 therapy induced polarization of M1 macrophages and participated in the antitumour immune response in a tumour-bearing mouse model. Treatment with a combination of OH2 and anti-SIRPα effectively inhibited tumour growth and significantly prolonged the survival time of the mice, and this result was more obvious in the mouse model with a larger tumour volume at the beginning of the treatment. These results suggest that combination therapy can more profoundly reshape the TME and activate stronger innate and adaptive immune responses.

**Conclusions:**

Our data support the feasibility of oncolytic virus therapy in combination with anti-SIRPα antibodies and suggest a new strategy for oncolytic virus therapy.

**Supplementary Information:**

The online version contains supplementary material available at 10.1186/s12916-022-02574-z.

## Background

The use of viruses for cancer therapy began more than a century ago. With the development of genetic engineering and advances in the understanding of the mechanism of action of viruses, oncolytic viruses (OVs) may become an ideal therapeutic platform. An increasing number of studies have shown that the killing effect of OVs on tumour cells is not only via direct cytolytic activity but also involves a complex regulatory mode combining multiple mechanisms [1]. These mechanisms include regulating changes in the tumour micro- and macroenvironment, specific immune responses mediated by CD8+ T cells, and innate immune cellular immune responses [2–4]. Despite their multiple mechanisms of therapeutic activity, many preclinical and clinical studies have shown that most oncolytic viruses, whether armed or unarmed, show limited efficacy as monotherapies [5, 6]. The ability of oncolytic viruses to modify the tumour microenvironment (TME) and alter immunologically "cold" tumours suggests that a combination of oncolytic viruses with other therapies, such as immunotherapy or chemotherapy, may achieve better therapeutic outcomes [7, 8].

At present, combining OVs and immune checkpoint blockade (ICB) is a research hotspot and has shown good efficacy in some clinical trials [9]. However, the combination of OVs and immunotherapy mainly focuses on the regulation of T cells, and there are few studies on its effect on innate immunity. OVs promote immunogenic cell death (ICD) during cell lysis, thereby recruiting and activating innate immune cells such as macrophages and dendritic cells through the release of damage-associated molecular patterns (DAMPs) and pathogen-associated molecular patterns (PAMPs) and further promoting the activation of tumour-specific T cells in the TME [4, 10]. Therefore, the activation of myeloid cells to activate tumour killing and enhance antigen presentation to activate endogenous immune function is expected to provide unique insights for antitumour therapy.

Macrophages are a class of highly plastic immune cells with a variety of functions that are usually divided into classically activated M1 macrophages and M2 macrophages based on their polarization state [11]. M1 macrophages promote the inflammatory Th1 response through the release of inflammatory cytokines and further enhance the T cell response through upregulation of antigen presentation and the expression of costimulatory molecules [11, 12]. Therefore, M1 macrophages may be involved in antitumour immunity in the tumour microenvironment. M2 macrophages are generally associated with the inhibition of endogenous antitumour immunity. Reducing the number of M2 macrophages and increasing the number of M1 macrophages are important prerequisites for successful tumour therapy. In addition, the phagocytic function of macrophages is regulated by the CD47-SIRPα antiphagocytic axis [13]. CD47 inhibits phagocytosis of macrophages through high expression on tumour cells [13, 14]. Anti-phagocytosis of the CD47-SIRPα axis can be blocked by antibodies, thus increasing the phagocytosis of macrophages. A recent study showed that blocking SIRPα on macrophages can effectively activate the antitumour ability of macrophages [15].

In previous studies [16, 17], we found that treatment with oncolytic herpes simplex virus 2 (OH2) can effectively alter the TME and induce an antitumour immune response in mice. In this study, we treated a mouse tumour model with a combination of OH2 and an anti-SIRPα antibody. We analysed the therapeutic effect and immune activation status of combination therapy. Our results suggest OH2 combined with the activation of macrophages is a promising antitumour therapy.

## Methods

### Cell line and oncolytic virus

The CT26, MC38, 4T-1, and RAW264.7 cell lines were purchased from the National Infrastructure of Cell Line Resource (Beijing, China) and kept by our laboratory. The cells were cultured in a constant temperature incubator containing 5% CO_2_ at 37°C.

OH2 was provided by Binhui Biopharmaceutical Co., Ltd. (Wuhan, China). The virus was an attenuated OH2 derived from the wild-type HSV-2 strain HG52 deleted the ICP47 gene and ICP34.5 gene [18, 19].

### Lysate preparation

The CT26, MC38, and 4T-1 cell lines in the logarithmic growth phase were passaged to 10 cm^2^ culture dishes when the cells reach 70–80% confluence, rinse the cells with PBS and then add 5 ml serum-free RPMI-1640 medium (HyClone, Waltham, MA) and infect the cells with OH2 according to MOI=1, and add 5 ml RPMI1640 medium containing 10% FBS (Gibco, Waltham, MA) after 1 h. After 30 h, the cell supernatant was collected, centrifuged at 4°C, 400 g for 5 min, and the lysate was collected and stored at −80°C for later use. The cell-free supernatant (CFS) of CT26, 4T-1, MC38, and untreated RAW264.7 were used as control. The CFS of CT26, 4T-1, and MC38 was obtained from the culture supernatant of cells in a logarithmic growth phase, centrifuged at 4°C, 400g for 5 min. The supernatant of CT26, MC38, and 4T1 cell lysate prepared by repeated freezing and thawing was also used as the control group.

### Cell viability test

RAW264.7 cells in the logarithmic growth phase were spread on a 96-well plate at 2000 cells/well and cultured for more than 6 h. After the cells adhered, added the lysate and controls were added to the cells, and Cell Counting Kit-8 (CCK8) detection was performed at the corresponding time points (0 h, 6 h, 12 h, 24 h, 48 h) (Dojindo, Kumamoto, Japan). Before detection, 100 μl of detection working solution (CCK8 reagent: RPMI1640 medium = 1:10) was added to each well and incubated for 1 h in a 37°C, 5% CO_2_ incubator in the dark. Finally, a microplate reader (Bio-Rad, Japan) was used to detect the absorbance of the cells at a wavelength of 450 nm.

### Flow cytometry to detect the polarization direction of macrophages

RAW264.7 cells in the logarithmic growth phase were spread to a 6-well plate at 10^6^ cells/well. After at least 6 h, the cells were completely attached to the wall, and the lysate and control were added separately. After 24 h of treatment, RAW264.7 cells were stained with FITC anti-mouse F4/80 (clone: FJK-16s, Invitrogen, Waltham, Massachusetts), APC anti-mouse CD86 (clone: GL-1, Biolegend, San Diego, CA) and PE anti-mouse CD206 (clone: MR6F3, Invitrogen, Waltham, Massachusetts) according to the protocol of the antibodies (M1 macrophage: F4/80+CD86+, M2 macrophage: F4/80+CD206+) and subjected to flow cytometry (LSR II, BD). Detection of SIRPα on macrophages in spleen from mouse used FITC anti-mouse F4/80, APC anti-mouse CD11b (clone: M1/70, Biolegend, San Diego, CA), and PE anti-mouse SIRPα (clone: P84, Biolegend, San Diego, CA). All flow cytometry used to detect macrophage typing in this study was set up with an antibody isotype control group (PE Rat IgG2a, clone: RTK2758, Biolegend, USA; APC Rat IgG2a, clone: RTK2758, Biolegend, USA; FITC Rat IgG2a, clone: RTK2758, Biolegend, USA).

### Functional experiments on macrophages

The CT26, MC38, and 4T-1 cell lines in the logarithmic growth phase were digested and resuspended at a concentration of 1×10^6^/mL. One μL of CFSE (C34554, Life Technology, Waltham, MA) working solution (0.5 mM) was added to every 2ml of tumour cells with a concentration of 1×10^6^/mL and incubated at 37°C (5% CO_2_) for 8 min. After the incubation, 10ml of pre-cooled RPMI 1640 medium containing 10% FBS was added to stop staining. The supernatant was discarded by centrifugation, and the cell concentration was adjusted to 5×10^5^/mL with RPMI 1640 medium with 10% FBS. The concentration of raw264.7 treated with lysate and control was adjusted to 5×10^6^/mL. With the ratio of 25:1, 50:1, and 100:1 as the effector to target ratio parallel samples, co-cultivation was carried out in a 96-well U-shaped well plate, 100μL raw264.7 cells, and 100μL tumour cells were added to each well, and 3 parallel samples were set up. Then placed the culture plate a 37°C (5% CO_2_) for 4 h. Before testing, 200 μL of PI (P8080, Solarbio, Beijing, China) working solution (2.5μg/mL) was added to each culture well.

### Detection of the expression level of CD47 and SIRPα in vivo and in vitro

The CT26, MC38, and 4T-1 cell lines and macrophages in the spleen of mice were stained with APC anti-mouse CD47 (clone: miap301, Biolegend, San Diego, CA), purified anti-mouse SIRPα (clone: P84, Biolegend, San Diego, CA) and anti-mouse IgG-Alexa 488 (ab150113, Abcam, Cambridge, UK) antibodies according to the protocol of the antibodies. The expression levels of CD47 and SIRPα were detected by flow cytometry.

### Immunohistochemical staining

The paraffin-embedded tissue sections were baked at 60–65°C for more than 6 h and then put in xylene for dewaxing while hot. Steps were followed by gradient ethanol hydration, citrate repair antigen (ZLI 9064, Zsjqbio, Beijing, China), 3% hydrogen peroxide blocking endogenous peroxidase activity, blocking (SP-KIT-B2, MXB, Fujian, China), primary antibody F480 (clone: SP115, dilution: 1:200, Abcam, Cambridge, UK), CD86 (clone: E5 W6H, dilution: 1:500, CST, Danvers, Massachusetts), CD206 (polyclonal, dilution: 1:100, Abcam, Cambridge, UK), CD8 (polyclonal, dilution: 1:200, Affinity Biosciences, Jiangsu, China), CD16 (polyclonal, dilution: 1:200, Affinity Biosciences, Jiangsu, China) and horseradish peroxidase (HRP)-conjugated secondary antibody (Kit-5010, MXB, Fujian, China) incubation, visualized with the 3,30-diaminobenzidine DAB (DAB-1031, MXB, Fujian, China) chromogen, haematoxylin staining (Solarbio, Beijing, China), hydrochloric acid ethanol differentiation, and ammonia water returning to blue. Finally, after dehydration with gradient ethanol, tissue sections were dehydrated in xylene and sealed with neutral gum. After drying, tissue sections were observed for staining under a microscope (Nikon Eclip se 80i, Japan).

### Multicolour immunohistochemical staining

The paraffin-embedded tissue sections were baked at 60°C-65°C for more than 6 h and then put in xylene for dewaxing while hot. After gradient ethanol hydration and neutral formalin immersion, each stained antibody was sequentially repaired by citrate, blocked by goat serum, and incubated with primary antibodies against F480 (clone: SP115, dilution: 1:500, Abcam, Cambridge, UK), CD86 (clone: E5 W6H, dilution: 1:1000, CST, Danvers, Massachusetts), CD206 (polyclonal, dilution: 1:200, Abcam, Cambridge, UK), CD8 (polyclonal, dilution: 1:500, Affinity Biosciences, Jiangsu, China), CD16 (polyclonal, dilution: 1:500, Affinity Biosciences, Jiangsu, China) and secondary antibody, and fluorescently stained to amplify the signal. After the staining was completed, the DAPI working solution was added dropwise, and finally, the super anti-quenching mounting tablet was added to the mount as required by the instructions in the kit (TSA-RM, PANOVUE, Beijing, China). The stained tissue slices were scanned and analysed with Plaris and Inform software.

IHC images were scanned using CaseViewer2.4 software. The degree of staining was scored: cells with staining <10% were scored as negative staining (−, 1), cells with staining 10–49% were scored as (+, 2); cells with staining rate of 50–74% were scored as (++,3), 75–100% stained cells were denoted as (+++, 4). Staining positive range scores are as follows: colourless (0); pale yellow particles (1), tan particles (2), and brown particles (3). The final score was defined as the staining extent score multiplied by the staining positive range score [20]. Negative expression scores ranged from 0 to 5, with positive expression scores over 5 [21].

### Animal model construction

The mice were purchased from Beijing Vital River Laboratory Animal Technology Co., Ltd. (Beijing Vital River Laboratory Animal Technology Co., Ltd.). Six- to 8-week-old female Balb/c mice weighing approximately 19–20 g were kept in a laminar flow ultraclean rack in our animal room under specific aseptic conditions for approximately 1 week. Each mouse was inoculated subcutaneously (s.c.) with 3×10^5^ CT26 cells on the right side of the dorsal area. The tumour appeared in approximately 5–7 days, and treatment was given when the diameter of the tumour grew to 3–5 mm or 8–10 mm.

All operations were carried out under the standard operating procedures of the specific pathogen-free (SPF) experimental mouse breeding management system specified by the state. All animal-related experimental procedures were approved by the Committee on the Ethics of Animal Experiments of the National Cancer Center/Cancer Hospital, Chinese Academy of Medical Sciences (CAMS), and Peking Union Medical College.

#### The first part

CT26 cell subcutaneous transplantation tumour model was constructed. When the diameter of the tumour reached 3–5 mm (day 7), the mice in the CL model were separated into three groups (OH2 [OH2 + Control Liposomes], OH2 + CL, and PBS control group) with an even distribution of tumour volumes. Assigned more than 10 mice to each group. OH2 (2×10^6^ plaque-forming units [PFU]) was performed by intratumoural injection (i.t.) on day 0, day 2, and day 4 in a volume of 100 μl. One hundred microliters of CL (7 mg/mL, F70101C-NC, FormuMax, Sunnyvale, CA) or control liposomes (7 mg/mL, F70101-N, FormuMax, Sunnyvale, CA) per mouse was given by intraperitoneal injection (i.p.) at the day before the OH2 treatment. The PBS control group was given 100 μL PBS per mouse. Tumour growth was regularly observed and recorded for each group of mice. After 14 days of treatment, the tumour tissues were resected for immunohistochemical and multicolour immunohistochemical staining. The mice in the survival observation group were tumour-bearing again on the 40th day.

#### The second part

A CT26 cell subcutaneous transplantation tumour model was constructed. When the diameter of the tumour reached 3–5 mm (day 7), it was defined as the tumour being smaller at the time of initial treatment. The mice in the anti-SIRPα model with smaller tumours at initial treatment were separated into six groups (OH2 + anti-SIRPα antibody group, OH2+isotype group, OH2 group, anti-SIRPα antibody group, isotype group, and PBS control group) with an even distribution of tumour volumes. Assigned more than 10 mice to each group. The OH2 treatment or PBS was injected on days 0, day 2, and day 4, and the anti-SIRPα antibody (clone: P84, Bioxcell, Lebanon, New Hampshire) or isotype (TNP6A7, Bioxcell, Lebanon, New Hampshire) was administered on days 1 and 3. The anti-SIRPα antibody and isotype were injected intraperitoneally (i.p.) with 100 μg, diluted to 1 mg/ml with PBS without preservation solution, and injected 100 μL intraperitoneally for each mouse. The PBS control group was given 100 μL PBS per mouse. The administration method and dosage of the OH2 were the same as those in the CL model. The tumour growth of mice was observed and recorded regularly every two days.

When the diameter of the tumour reached 8–10 mm (day 14), it was defined as the tumour being larger at the time of initial treatment. The mice in the anti-SIRPα model with larger tumours at initial treatment were separated into four groups (OH2+ anti-SIRPα antibody group, OH2+isotype group, OH2 group, anti-SIRPα antibody group, and PBS control group) with an even distribution of tumour volumes. Assigned more than 10 mice to each group. The mode of administration and treatment strategy was the same as described above. The tumour growth of mice was observed and recorded regularly. After 12 days of treatment, the tumour tissues were resected for immunohistochemical, multicolour immunohistochemical staining, and RNA sequencing.

When the experimental endpoint or humanitarian endpoint was reached, such as when the size of the mouse reached 2500 mm^3^ or tumour metastasis or rapid growth caused ulceration, necrosis, or infection that interfered with eating or walking, the mice were anaesthetized with 5% chloral hydrate and then sacrificed by cervical dislocation. The calculation formula of tumour volume was volume = (length×width^2^)/2.

### RNA sequencing

The transcriptome sequencing involved in this study was undertaken and completed by Tianjin Nuohe Zhiyuan Bioinformation Technology Co., Ltd. RNA samples were required to reach a total volume of ≥30 μL, a total volume of ≥1.5 μg, and a concentration of ≥50 ng/μL. The agarose gel electrophoresis quantification method, Nanodrop, and Agilent 2100 were used to test the concentration, purity, and integrity of the submitted RNA. The type of library construction was a eukaryotic chain-specific library. The sequencing strategy was Illumina Hiseq-PE150 (two-way sequencing).

### Single-cell RNA sequencing

The tumour tissues of the control group, OH2 group, and combined treatment group were subjected to single-cell sequencing analysis, and the single-cell sequencing technology used in this study was provided by Huada Company. The tissue was dissociated using the Tumor Dissociation Kit (mouse, MACS), the dead cells were removed, and the single-cell suspension of living cells was left for sequencing. The data obtained were analysed using the Seurat package, and the volcano plot was drawn using the R software package EnhancedVolcano. The R packages used for GO analysis and KEGG analysis included tidyverse, patchwork, monocle, clusterProfiler, org.Mm.eg.db.

### Bioinformatics analysis

R software version 4.0.2 was used to analyse the transcriptome sequencing data, and the R software package “edgeR” was used for differential expression analysis between groups. Gene set enrichment analysis (GSEA) was used to study the difference signal pathways between different groups. The R software package “clusterProfiler” was used for Gene Ontology (GO) and Kyoto Encyclopedia of Genes and Genomes (KEGG) analysis. Gene set variation analysis (GSVA) was used for immune cell infiltration analysis. Student’s *t*-test was used to compare the differentially expressed genes, and the *P*-value was adjusted and corrected by the Benjamini-Hochberg method. The log2-fold-change was greater than 1, and the corrected *P*-value was less than 0.01 as the threshold to judge the significance of the difference. A total of 770 immunology-related mouse genes created from the nCounter Mouse PanCancer Immune Profiling Panel (NanoString) were used as reference genes which are listed in Additional file 1: Table S1 and Additional file 2: Table S2.

### Statistical analysis

GraphPad Prism software version 8 was used for statistical analysis and statistically significant differences were defined as a *p* value<0.05. Two-tailed, unpaired Student’s *t*-test was used to compare two groups. Two-way analysis of variance (ANOVA) was performed on the experimental data for tumour volumes for more than two groups. The Kaplan-Meier method and log-rank test were used for the survival curve data; the survival period was defined as the time from the start of treatment to the end of the observation. The results are presented as the mean ± S.E.M. In this study, all experiments were repeated at least three times, except for the parts otherwise stated.

## Results

### OH2 lysates could induce macrophage polarization and activation in vitro

**Fig. 1:**
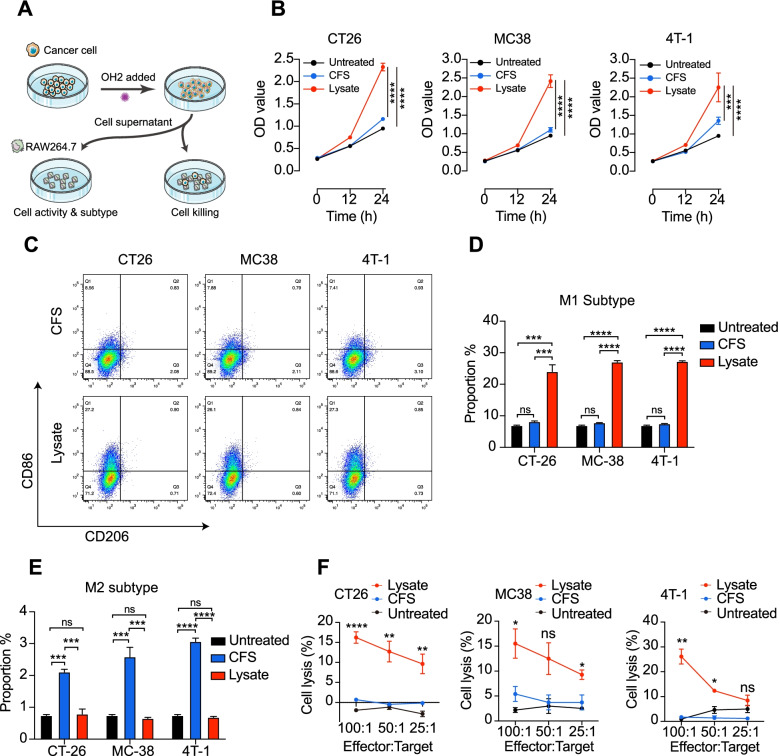
OH2 lysates induce RAW264.7 polarization and phagocytosis in vitro. **A** Schematic of lysate preparation and flowchart of the experiment performed in the study. **B** Cell proliferation assay results of CT26 (left), MC38 (middle), and 4T-1 (right) cell lines treated with lysate (red), CFS (blue), and untreated (black) group by CCK8 assay in 24 h. **C** Flow cytometric analysis results of one representative sample from each cell line with lysate or CFS treatment. **D** The percentage of M1 (F4/80+CD86+) subtype in the lysate, CFS, and untreated groups of CT26, MC38, and 4T-1 cells detected by flow cytometry. The data are averages from three samples per treatment group. An unpaired Student’s *t* test was used to analyse the significance of the difference between the two groups and ANOVA was used to analyse the significance of the difference between the groups (>2). **E** The ratio of M2 (F4/80+CD206+) subtype in the lysate, CFS, and untreated groups of CT26, MC38, and 4T-1 cells detected by flow cytometry. The data are averages from three samples per treatment group. An unpaired Student’s *t* test was used to analyse the significance of the difference between the groups. **F**. The phagocytic and killing functions of macrophages treated with different cancer cell lysates detected by CFSE/PI. Three effectors to target ratios were set for each cell line (RAW264.7: tumour cells=25:1, 50:1, and 100:1). The data are averages from three samples per treatment group. Statistical analysis was performed using ANOVA with multiple comparisons. ns, no significant differences, *, *p*<0.05, **, *p*<0.01, ***, *p*<0.001, ****, *p*<0.0001

We confirmed the role of macrophages in the process of OH2 treatment of colon cancer, as shown in Fig. 1A. As shown in Fig. 1B, the RAW264.7 cells treated with the lysates had significantly enhanced viability and proliferation ability by CCK8 assay (*p*<0.0001 for CT26 and MC38 cells, *p*=0.001 and *p*<0.0001 compared with the cell-free supernatant (CFS) and untreated RAW264.7 and 4T-1 cells, respectively). The lysates stimulated the activation of macrophages. We also incubated OH2 at different MOIs (MOI=1, MOI=0.5) with RAW264.7 cells to determine whether OH could directly activate macrophages through CCK8. As expected, the addition of OH2 (Additional file 3: Fig. S1) or frozen cell lysate (Additional file 3: Fig. S2) could not activate macrophages directly within 24 h.

Subsequently, we used flow cytometry to detect the ratio of M1 (F4/80+CD86+) and M2 (F4/80+CD206+) macrophages after lysate treatment (Fig. 1C). The proportion of M1 macrophages after lysate and CFS treatment significantly increased (*p*=0.0015, *p*<0.0001, and *p*<0.0001 for CT26, MC38 and 4T-1 lysate treatment, respectively, and *p*=0.01, *p*=0.0073, and *p*=0.023 for CT26, MC38, and 4T-1 CFS treatment, respectively) compared with the untreated group (Fig. 1D). There were also significant differences between the lysates and CFS treatment (*p*=0.01, *p*=0.0018, and *p*<0.0001 for CT26, MC38, and 4T-1 lysate treatment, respectively). Interestingly, there was no difference in the proportion of M2 macrophages between the lysate and untreated groups, but the proportion of M2 macrophages in the CFS increased significantly compared with that in the other two groups (Fig. 1E, Additional file 3: Fig. S3). The ratio of M1 (F4/80+CD86+) and M2 (F4/80+CD206+) macrophages in RAW264.7 cells treated with lysate in the blocked SIRPα group and the nonblocked SIRPα group was not significantly different (*p*=0.010, Additional file 3: Fig. S4). The difference in RAW264.7 polarization induced by frozen cell lysate and CFS was not statistically significant (Additional file 3: Fig. S5). Similar results were also observed in mouse splenic primary macrophages. CFS did not significantly change the proportion of M1 or M2, while the proportion of both M1 and M2 in primary macrophages increased significantly after lysate treatment (*p*<0.0001). Although frozen cell lysate also increased the proportion of M1, it was more reflected in the proportion of M2 (Additional file 3: Fig. S6). These results indicated that the lysates effectively stimulated macrophages to polarize towards the M1 but not the M2 phenotype. To uncover the relationship between macrophage polarization and phagocytosis, a cell killing assay was performed. There were no significant differences between the CFS and untreated groups. Lysate-polarized M1 macrophages had a significant killing effect compared with the other two groups, and the effect was enhanced with an increase in the effector cell:target cell ratio (E:T) (Fig. 1F).

**Fig. 2:**
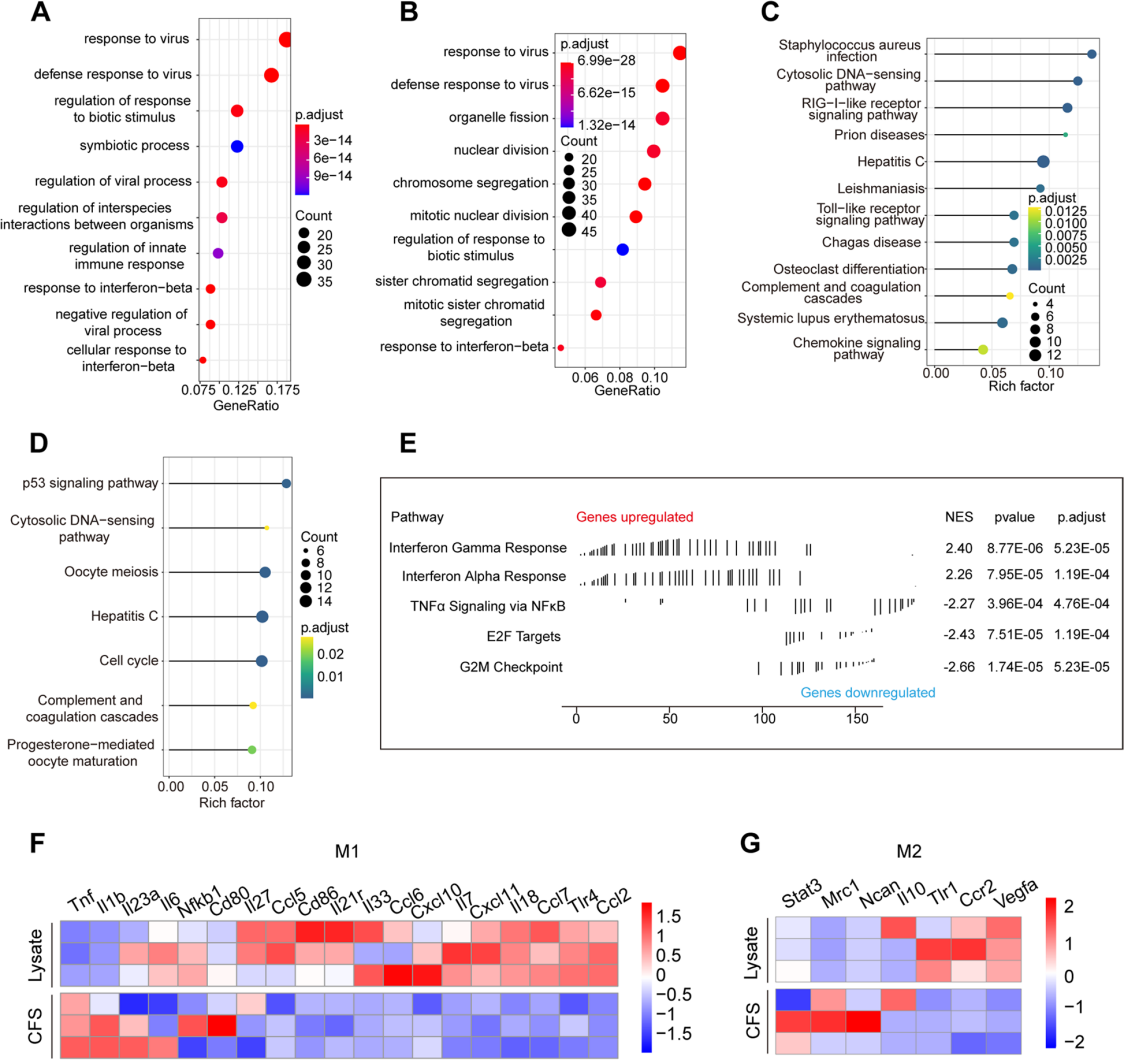
RNA sequencing of lysate-treated, CFS-treated, and untreated groups characterized Raw264.7 function. **A** GO analysis of differentially expressed genes of RAW264.7 cells treated with lysate and CFS. **B** GO analysis of differentially expressed genes of RAW264.7 cells treated with lysate and untreated RAW264.7 cells. **C** KEGG analysis of differentially expressed genes of RAW264.7 cells treated with lysate and CFS. **D** KEGG analysis of differentially expressed genes of RAW264.7 cells treated with lysate and untreated RAW264.7 cells. **E** GSEA of differentially expressed genes of RAW264.7 cells treated with lysate and CFS. **F** The expression of M1 macrophage markers in RAW264.7 cells treated with lysate and CFS. **G** The expression of M2 macrophage markers in RAW264.7 cells treated with lysate and CFS. The colour represents the value of the expression level of the gene in RNA sequencing

To explore the changes in macrophages with polarization, RNA sequencing was performed on different RAW264.7 treatment groups. Whether CT26 lysates were compared with supernatant or untreated RAW264.7 cells, GO analysis showed that the lysates activated the activity of antiviral signalling pathways in RAW264.7 cells, including response to virus, regulation of viral process, regulation of innate immune response, and response to interferon-beta (Fig. 2A and B). KEGG analysis showed the same signalling changes as lysate treatment (Fig. 2C and D). Furthermore, GSEA showed that interferon-related pathways were upregulated and cell proliferation-related signalling pathways were downregulated after lysate treatment (Fig. 2E). Compared with the supernatant group, the expression of M1 markers (Il23a, Il6, Nfkb1, Cd80, Il27, Ccl5, Cd86, Il21r, Il33, Ccl6, Cxcl10, Il7, Cxcl11, Il18, Ccl7, Tlr4, and Ccl2) in the lysate treatment group was upregulated, and the expression of M2 markers (Stat3, Mr1, and Ncan) was downregulated (Fig. 2F and G). These results suggested that lysate treatment could effectively induce polarization of M1 macrophages, activate the function of M1 macrophages, and activate antiviral and antitumour immune responses.

### OH2 treatment polarized macrophages to the M1 phenotype for tumour killing in vivo

**Fig. 3:**
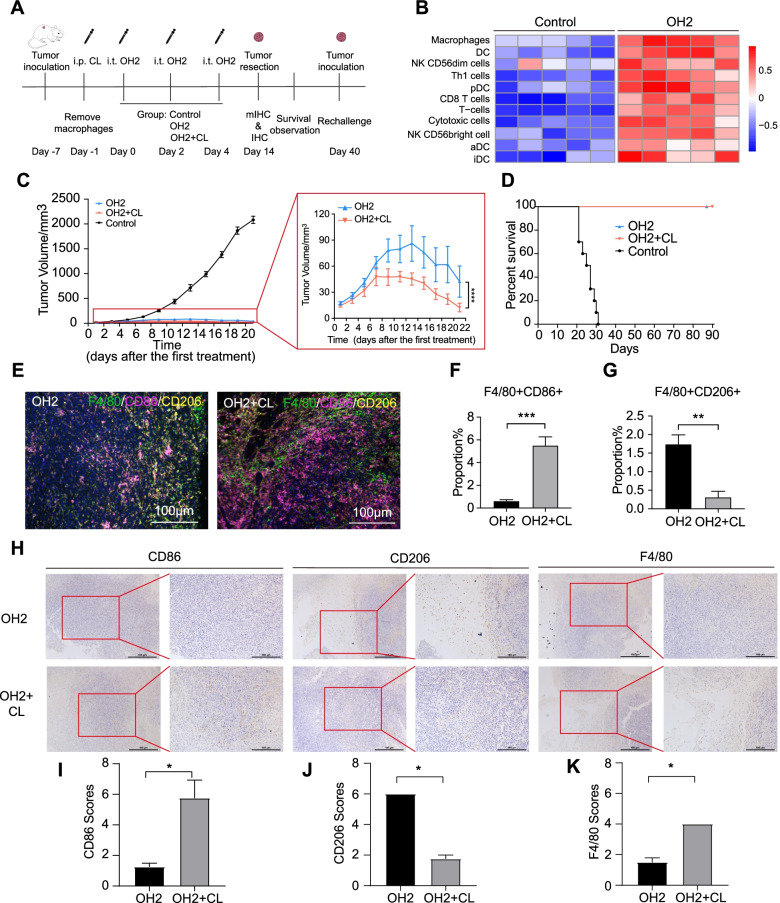
OH2 treatment polarized macrophages to the M1 phenotype for tumour killing in vivo. **A** The treatment process and experiment timeline of the CL animal model. **B** The immune cell infiltration analysis results of the OH2 treatment group and control group by GSVA. **C** The tumour growth curve of the OH2 combined CL group (red), OH2 (OH2 with control liposomes) group (blue), and control group (black). The red box zoomed (right) in to show the OH2 combined CL group and OH2 group. Two-way analysis of variance (ANOVA) was performed on the experimental data. *n*=10 mice/group, ****, *P*<0.0001. **D** The survival curve of the OH2 combined CL group (red), OH2 group (blue), and control group (black). The Kaplan-Meier method and log-rank test were used for the survival curve data. ns, no significant differences. **E** Representative M-IHC results of the OH2 group (left) and OH2 combined CL group (right). F4/80, green, CD86, purple, and CD206, yellow. Scale bar, 100μm. **F** M-IHC quantification results for M1 subtype. *n*=3 samples/group. The Student’s *t* test was used to analyse the significance of the difference between the two groups. ***, *p*<0.001. **G** M-IHC quantification results for M2 subtype. *n*=3 samples/group. The Student’s *t* test was used to analyse the significance of the difference between the two groups. **, *p*<0.01. **H** Representative IHC staining for CD86 (left), CD206 (middle), and F4/80 (right) in the OH2 (top) and OH2 combined CL group (bottom). Original magnification, ×200. *n*=5 samples/group. (Percentage represented the proportion of M1/M2 macrophages in the total number of cells). **I**. Quantitative results of CD86 staining in the OH2 combined CL and OH2 group. **J** Quantitative results of CD206 staining in the OH2 combined CL and OH2 group. **K** Quantitative results of F4/80 staining in the OH2 combined CL and OH2 group. *n*=3 samples/group. The Student’s *t* test was used to analyse the significance of the difference between the two groups. ns, no significant differences, *, *p*<0.05, **, *p*<0.01, ***, *p*<0.001, ****, *p*<0.0001

We explored the correlation between OH2 treatment and macrophage function in vivo, as shown in Fig. 3A. Based on previous research [16], we showed that OH2 therapy promoted the infiltration of adaptive immune cells (T cells) and innate immune cells (macrophages, DCs and NK cells) (Fig. 3B).

Macrophages in CT26-bearing mice were cleared using clodronate liposomes (CLs) one day before treatment. First, we verified the clearance efficiency and found it reached more than 90% within 24 h after the injection and then recovered and was higher than the preinjection level 72 h later (Additional file 3: Fig. S7). These results suggest that the use of CL alone can effectively remove any existing macrophages without affecting the formation of new macrophages.

As shown in Fig. 3C, both OH2 therapy and CL combined with OH2 therapy showed strong tumour inhibition. Indeed, the combination therapy showed earlier tumour suppression and a more significant therapeutic effect (*p*<0.0001) (Fig. 3C). However, there was no difference in survival between the two groups, and all of the tumours in the combined and OH2 groups ultimately regressed (Fig. 3D). The rechallenge experiment showed that the tumour formation rate of the combined group was significantly lower than that of the OV group (*p*=0.029), although the tumours eventually returned (Additional file 3: Fig. S8). This implies that macrophages might play a role in the treatment effect of OH2.

Quantitative analysis by multicolour immunohistochemistry (M-IHC) showed that the combined use of CL and OH2 promoted an increase in M1 macrophages (*p*=0.0004) in the tumour immune microenvironment, accompanied by a decline in M2 macrophages (*p*=0.0011) compared with OH2 alone (Fig. 3E–G, Additional file 3: Fig. S9). The immunohistochemistry results showed that the M1 macrophage marker was highly expressed in tumour tissues, and the M2 macrophage marker was only expressed at low levels in the combination therapy group (Fig. 3H–K). These results suggested that OH2 therapy can induce polarization of M1 macrophages and participate in the antitumour immune response in vivo.

### Combined anti-SIRPα therapy with OH2 enhances anticancer immune responses in a CT26 cancer model

**Fig. 4:**
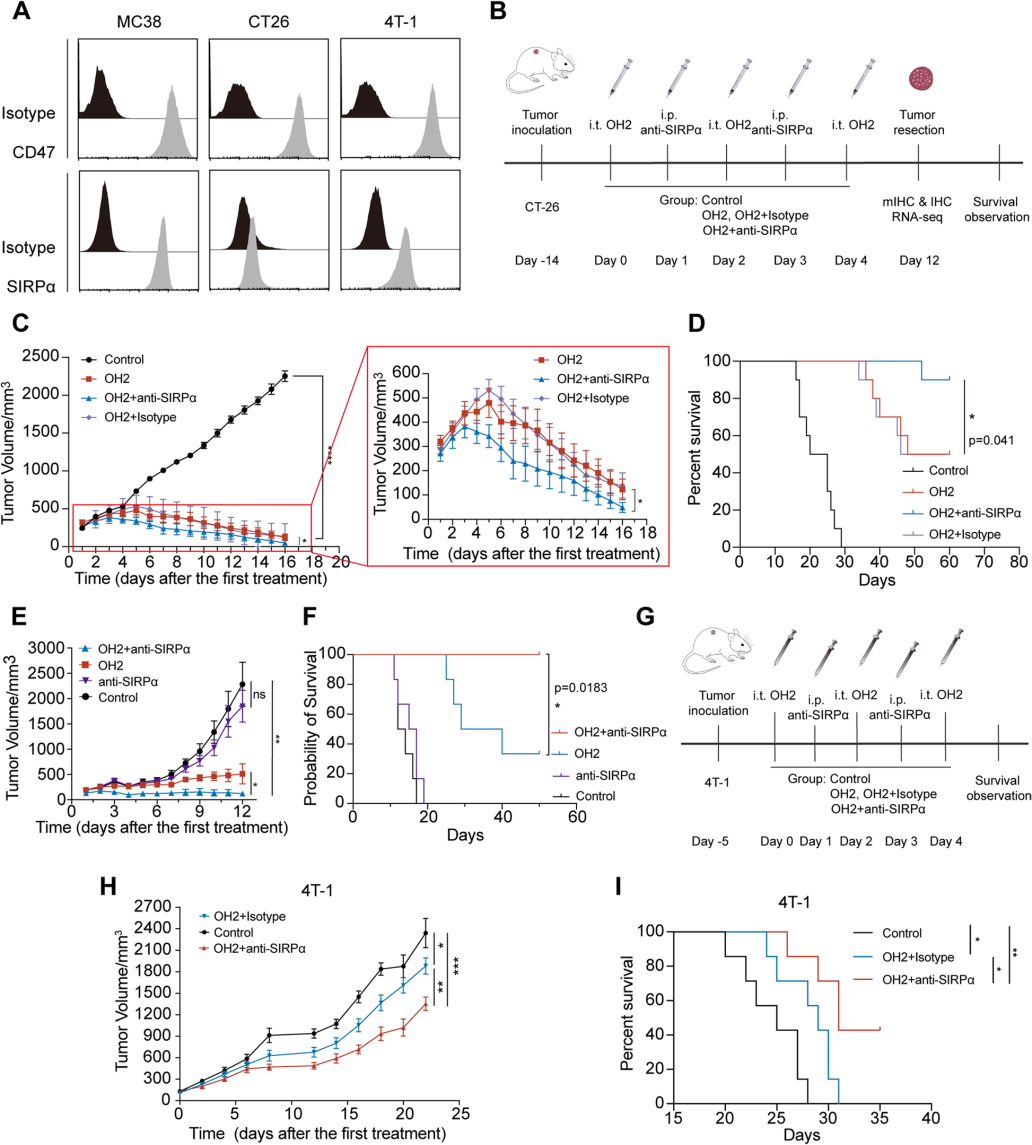
The combination of OH2 and anti-SIRPα therapy enhances anticancer immune responses in vivo. **A** The expression levels of CD47 and SIRPα in CT26, MC38, and 4T-1 cell lines were detected by flow cytometry. **B** The treatment process and the experimental timeline for adjusted combined treatment. **C** The tumour growth curve of different treatment groups of tumour-bearing mice with larger tumour volumes at the initial treatment. The red box zoomed in to show the OH2 combined anti-SIRPα antibody group, OH2 combined isotype, and OH2 group. Two-way analysis of variance (ANOVA) was performed on the experimental data. *n*=10 mice/group. *, *p*=0.019. **D** The survival curve of different treatment groups of tumour-bearing mice with larger tumour volumes at the initial treatment. The Kaplan-Meier method and log-rank test were used for the survival curve data. *, *p*=0.041. **E** The tumour growth curve of the OH2 combined anti-SIRPα antibody group, anti-SIRPα antibody group, OH2 group, and control group. Two-way analysis of variance (ANOVA) was performed on the experimental data. *n*=6 mice/group. *; **. **F** The survival curve of the OH2 combined anti-SIRPα antibody group, anti-SIRPα antibody group, OH2 group, and control group. The Kaplan-Meier method and log-rank test were used for the survival curve data. *, *p*=0.0183. **G** The treatment process and experimental timeline for the combined treatment. **H** The tumour growth curve of the OH2 combined anti-SIRPα antibody group, OH2 combined isotype, and control group. Two-way analysis of variance (ANOVA) was performed on the experimental data. *n*=7 mice/group. *, *p*=0.029; **, *P*=0.0041; ***, *p*=0.0003. **I** The survival curve of the OH2 combined anti-SIRPα antibody group, OH2 combined isotype, and control group. The Kaplan-Meier method and log-rank test were used for the survival curve data. *, *p*=0.03 and *p*=0.018; **, *P*=0.0014

To further explore the feasibility of OH2 combined with macrophage therapy, an anti-SIRPα antibody that blocks the CD47-SIRPα axis in macrophages was used. First, we detected the expression of CD47 and SIRPα in cell lines. As shown in Fig. 4A, CD47 was widely expressed in most cell lines, while SIRPα expression was cell-line specific. The expression level of SIRPα on macrophages in the spleens of mice was shown in Additional file 3: Fig. S10. These results suggested that we could remove the blocking effect of CD47 on macrophage phagocytosis with an anti-SIRPα antibody.

An in vivo test was performed. When the CT26 tumour volume was small at the start of treatment (45.315±4.403 mm^3^), the tumour volume and survival were not significantly different between the combined treatment group and the OH2 treatment group (*p*=0.4712) (Additional file 3: Fig. S11). Whether as a single or combined treatment, OH2 could exert excellent antitumour efficacy, and the tumour would eventually regress completely. Then, we adjusted the in vivo experiment (Fig. 4B). We started treatment on day 14 when the tumour was larger (281.596±31.469 mm^3^), and a difference in efficacy between the OV treatment group and the combined treatment group began to appear (Fig. 4C). Compared with the other groups, the tumours in the combined treatment group regressed faster. In addition, mice in the combination group had a higher survival rate (*P*=0.041) than mice in the OH2 and OH2+iIsotype groups (Fig. 4D). The number of mice that achieved tumour regression was also the largest. Compared with the control group, both groups using OH2 were able to effectively inhibit tumour growth and prolong the survival of the mice (Fig. 4E, F). However, the anti-SIRPα antibody alone neither inhibited tumour growth (*P*>0.05, Fig. 4E) nor prolonged the survival time of tumour-bearing mice (*P*>0.05, Fig. 4F).

We also verified immune exclusion in 4T-1 mouse models (Fig. 4G). As shown in Fig. 4H, the combination group showed a potent antitumour effect that was significantly different from the OH2 group (*p*=0.0041) and the control group (*p*=0.003). In addition, mice in the combination group had a better survival rate than mice in the OH2 (*p*=0.03) and control groups (*p*=0.0014) (Fig. 4I). These results showed that the anti-SIRPα antibodies enhanced the efficacy of OH2 therapy.

**Fig. 5:**
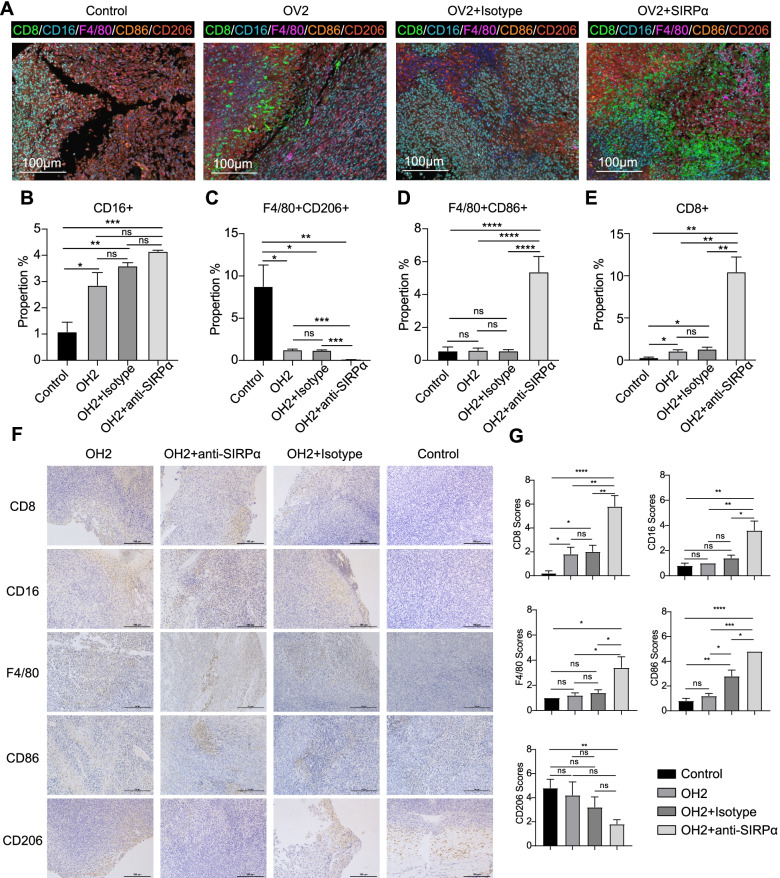
M-IHC and IHC staining of the anti-SIRPα antibody treatment model. **A** Representative M-IHC results of the OH2 combined anti-SIRPα antibody group, OH2 combined isotype, OH2 group, and control group with larger tumour volume sat the initial treatment. CD8, green, CD16, sky blue, F4/80, purple, CD86, orange, and CD206, red. Scale bar, 100μm. B Quantitative results of NK cell (CD16) staining in the OH2 combined anti-SIRPα antibody group, OH2 combined isotype, OH2 group, and control group. *n*=3 samples/group. **C** Quantitative result of M2 macrophage (F4/80+CD206+) staining in the OH2 combined anti-SIRPα antibody group, OH2 combined isotype, OH2 group, and control group. *n*=3 samples/group. **D** Quantitative results of M1 macrophage (F4/80+CD86+) staining in the OH2 combined anti-SIRPα antibody group, OH2 combined isotype, OH2 group, and control group. *n*=3 samples/group. **E** Quantitative results of CD8 T cell staining in the OH2 combined anti-SIRPα antibody group, OH2 combined Isotype, OH2 group, and control group. *n*=3 samples/group. **F** Representative IHC staining for CD8, CD16, F4/80, CD86, and CD206 of the OH2 combined anti-SIRPα antibody group, OH2 combined isotype group, OH2 group, and control group with larger tumour volumes at the initial treatment. Original magnification, ×200. *n*=5 samples/group. **G** Quantitative results of CD8, CD16, F4/80, CD86 and CD206 staining in the OH2 combined anti-SIRPα antibody group, OH2 combined isotype, OH2 group and control group. *n*=5 samples/group. Statistical analysis was performed using ANOVA with multiple comparisons. ns, no significant differences, *, *p*<0.05, **, *p*<0.01, ***, *p*<0.001, ****, *p*<0.0001 (proportion represented the percentage of the cell to the total number of cells)

Subsequently, we performed a pathological analysis of the tumour tissue from the mice. The pathological results showed that in the tumour microenvironment of mice that did not receive treatment, the proportion of M2 macrophages was much higher than that of M1 macrophages (Fig. 5A, Additional file 3: Fig. S12). Treatment with OH2 reduced the infiltration of M2 macrophages (*p*<0.001) and increased the infiltration of NK cells (*p*<0.05) into the tumour microenvironment to a certain extent (Fig. 5B and C). The combined use of anti-SIRPα antibody and OH2 resulted in increased infiltration of CD8 T cells (*p*<0.01) and M1 macrophages (*p*<0.001) into the tumour microenvironment (Fig. 5D and E). The immunohistochemistry results showed that the immune cells of the combined treatment group were concentrated in the central part of the tumour (Fig. 5F, G and Additional file 3: Fig. S13). This implied that the ability of these tumour-inhibiting immune cells to migrate to the centre of the tumour was enhanced. These results suggested that combination therapy can stimulate a stronger antitumour immune response than treatment alone.

### Combination therapy reshapes the TME and activates a more comprehensive antitumour immune response.

**Fig. 6:**
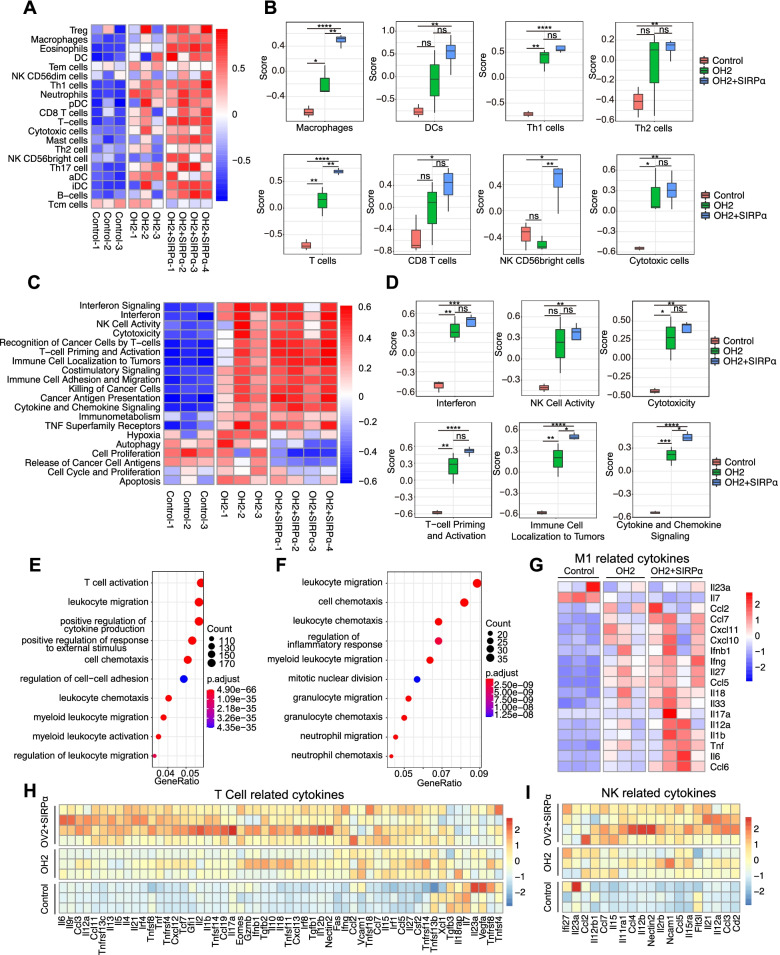
The tumour immune microenvironment revealed by RNA sequencing results of tumour tissues in different treatment groups. **A** The immune cell infiltration analysis results of tumour tissues from the OH2 combined anti-SIRPα antibody group, OH2 group, and control group by GSVA. **B** The scoring results of immune cells (macrophages, DCs, Th1 cells, Th2 cells, T cells, CD8 T cells, NK CD56bright cells, and cytotoxic cells) of tumour tissues from the OH2 combined anti-SIRPα antibody group, OH2 group, and control group by GSVA. **C** The immune-related signalling pathways of tumour tissues from the OH2 combined anti-SIRPα antibody group, OH2 group, and control group by GSVA. **D** The scoring results of immune-related signalling pathways (interferon, NK cell activity, cytotoxicity, T cell priming, and activation, immune cell localization to tumours, cytokine, and chemokine signalling) of tumour tissues from the OH2 combined anti-SIRPα antibody group, OH2 group, and control group by GSVA. **E** GO analysis of differentially expressed genes in the control group and the OH2 combined anti-SIRPα antibody group. **F** GO analysis of differentially expressed genes in the OH2 group and the OH2 combined anti-SIRPα antibody group. **G** The expression level of M1-related cytokines in the OH2 group, OH2 combined with anti-SIRPα antibody group and control group. **H** The expression levels of T cell-related cytokines in the OH2 group, and OH2 combined with the anti-SIRPα antibody group and the control group. **I** The expression level of NK-related cytokines in the OH2 group, OH2 combined with anti-SIRPα antibody group and control group. An unpaired Student’s *t* test was used to analyse the significance of the difference between the two groups. ns, no significant differences, *, *p*<0.05, **, *p*<0.01, ***, *p*<0.001, ****, *p*<0.0001. The colour represents the value of the expression level of the gene in RNA sequencing. OH2 + SIRPα represents the OH2 combined with the anti-SIRPα antibody group

We used RNA sequencing and single-RNA sequencing to characterize the TME in more detail. OH2 treatment alone can promote the infiltration of adaptive immune cells (T cells and Th1 cells) and innate immune cells (macrophages and DCs) into the tumour immune microenvironment (Fig. 6A). In the combination therapy group, the scores of macrophages were much higher. Although there was no significant difference, DCs, CD8+, and cytotoxicity were improved by the combination compared to OH2 treatment alone (Fig. 6B). When anti-SIRPα and OH2 were combined, they were more conducive to the infiltration of immune cells into tumour tissues, especially macrophages, DCs, T cells, and NK cells. Immune cells were globally activated, thereby creating an antitumour tumour microenvironment (Fig. 6C). As a result of immune cell infiltration, the antigen presentation signal in the tumours of the combined treatment group was enhanced, and the killing function of the immune cells was strengthened (Fig. 6D, and Additional file 3: Fig. S14). However, the proliferation ability of the tumour cells decreased, and apoptosis increased (Fig. 6C, Additional file 3: Fig. S15). The GO analysis showed that leukocyte migration and chemotaxis were enhanced in the combined treatment group, consistent with the IHC results (Fig. 6E and F). The enhanced migration ability and functional activation of immune cells in tumour tissues, as well as the full activation of M1-related cytokines, T cell-related cytokines, and NK-related cytokines, promoted the elimination of tumour cells (Fig. 6G–I). These results suggested that combination therapy can more profoundly reshape the TME and activate stronger innate and adaptive immune responses.

**Fig. 7:**
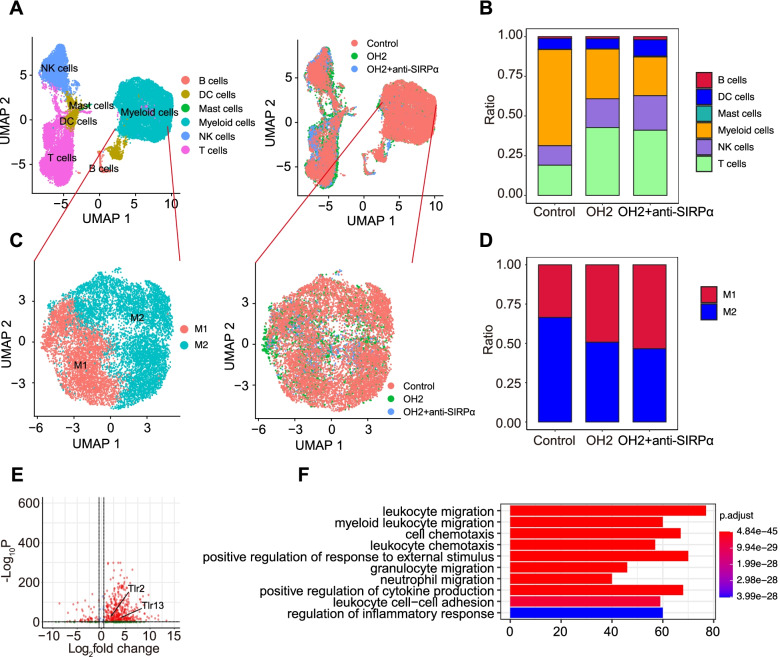
The tumour immune microenvironment revealed by Single-RNA sequencing results in tumour tissues in different treatment groups. **A** Clustering of immune cells in single-cell data. **B** Proportions of different subsets of immune cells in single-cell data. **C** Clustering of macrophages in single-cell data. **D** Proportions of different subsets of macrophages in single-cell data. **E** Volcano plot showing toll-like receptors in differentially expressed genes in the OH2 combined anti-SIRPα antibody group. **F** GO analysis of differentially expressed genes in the OH2 combined anti-SIRPα antibody group

The single-cell data showed that immune cells in the tumour microenvironment could be divided into T cells, B cells, NK cells, myeloid cells, mast cells, and DC cells (Fig. 7A, and Additional file 3: Fig. S16). The proportion of each immune cell subset is shown in Fig. 7B. Compared with the control group, the T cells, NK cells and DC cells in the OH2 group and the OH2 combined anti-SIRPα antibody group increased, while the myeloid cells decreased. Compared with the group treated with OH2 alone, NK cells and DC cells were significantly increased in the combination treatment group, and myeloid cells were significantly decreased (Fig. 7B). Analysis of the different types of macrophages showed that (Fig. 7C) M1 macrophages were increased in both groups treated with OH2, but the increase in M1 macrophages was more significant in the combined treatment group (Fig. 7D). The genes differentially expressed between the OH2 combined anti-SIRPα antibody group and the two other groups were identified. Virus nucleic acid fragments, unpackaged intact capsid proteins, or cellular contents might be involved in the treatment as PAMPs/DAMPs. The results of single-cell sequencing data showed that Tlr2 and Tlr13 are upregulated (Fig. 7E). Tlr2 and Tlr13 recognize virus components and activate Toll-like receptor (TLR)-dependent signalling pathways [22, 23], and the function of immune cells was strongly activated (Fig. 7F).

To verified the tumour-specific cytotoxic T lymphocyte (CTL) response of treatment, the spleen lymphocytes isolated from different treatment groups (control, anti-SIRPα, OH2, and OH2+ anti-SIRPα) were co-cultured with CT26 cells in vitro at target cell/effector cell (T:E) ratios of 1:100, 1:50, and 1:25. Lymphocytes from both the OH2 group and the OH2+ anti-SIRPα group showed a highly specific CTL response against CT26 cells (*p*<0.001). The CTL response of OH2 combined with anti-SIRPα antibody was more intense than that of OH2 alone (*p*<0.05). However, no CTL response was detected with anti-SIRPα antibody alone (Additional file 3: Fig. S17A). ELISA was used to detect the supernatant of co-cultured cells, and we found that the expression of TNF-α, Granzyme B and IFN-γ could be significantly induced in the OH2 group and the OH2+anti-SIRPα group (*p*<0.0001, Additional file 3: Fig. S17B). Overall, T cell effector cytokines were slightly higher in the OH2+anti-SIRPα group than in the OH2 group, although there was no statistical difference. Also, T cell effector cytokines were not detected with anti-SIRPα antibody alone. These results suggested that combination therapy can also improve the tumour-specific CTLs.

## Discussion

**Fig. 8:**
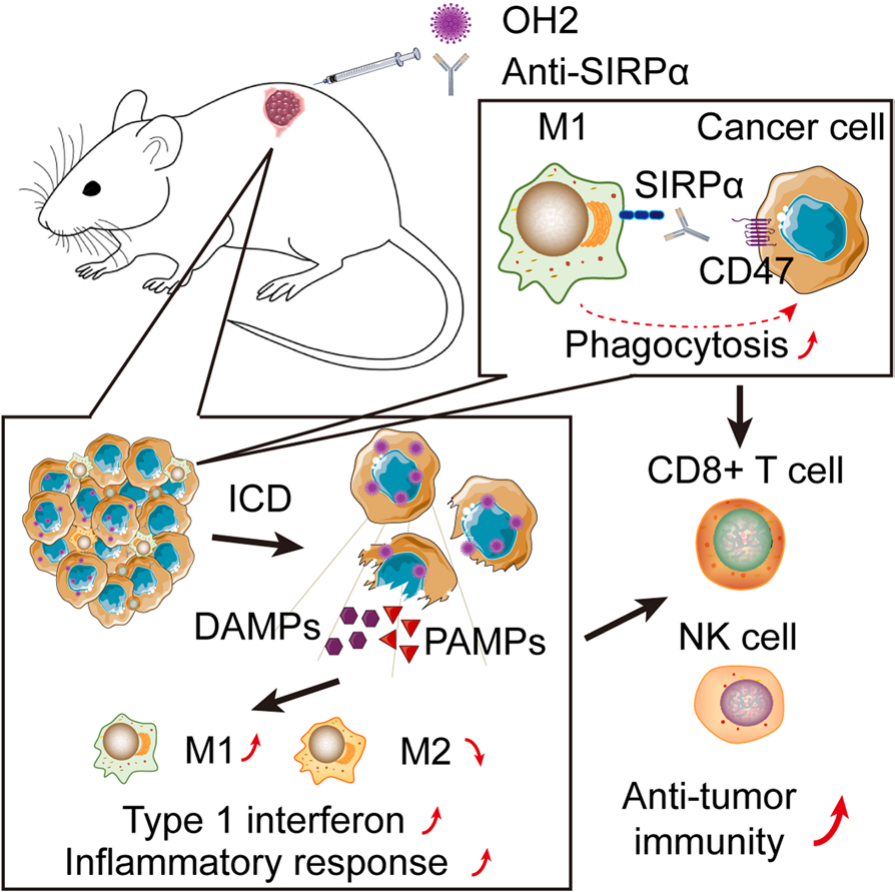
OH2 treatment polarized macrophages to M1 for tumour killing in vivo, and the SIRPα antibody combined with OH2 therapy reshapes the TME and activates a more comprehensive anti-tumour immune response. The introduction of the SIRPα antibody can accelerate cellular reconstitution of TME and induce a specific antitumour immune response through an earlier innate immune response

We report the efficacy and molecular and immunological effects of combination therapy with oncolytic herpes simplex virus and anti-SIRPα in a mouse model in vitro and in vivo (Fig. 8). We demonstrated that tumour cell lysate-induced OH2 can effectively activate and induce the differentiation of mouse RAW264.7 macrophages towards the M1 phenotype, enhancing their phagocytosis and killing effects on tumour cells in vitro. We observed that the combination of OH2 and anti-SIRPα, which blocks the CD47-SIRPα axis, can effectively enhance the therapeutic effect by enhancing the innate immune effect of macrophages. The combination of OH2 and anti-SIRPα also showed stronger regulation of the tumour immune microenvironment.

In recent years, OVs have been considered a promising new strategy for cancer treatment. Compared with surgical therapy, chemoradiotherapy and targeted therapy, OVs have shown high killing ability, precise targeting ability, and few side effects or drug resistance [9, 24, 25]. OVs can be genetically modified to enhance their tumour targeting ability, improve their safety, and increase their antitumour efficacy [26, 27]. More than 40 OV types are currently being evaluated in clinical trials for the treatment of various tumours, most of which are in phase I studies [24], but only T-VEC has successfully entered phase III clinical trials and received marketing approval [28].

The OH2 used in the current study showed good intratumoural injection tolerance and persistent antitumour activity in patients with metastatic oesophageal and rectal cancer in a recent study [29]. Although a large number of clinical trials have shown positive results with OVs, their efficacy as monotherapy agents is limited [6, 30, 31]. Therefore, combination therapy is one of the strategies used to enhance the therapeutic effect of OVs [32, 33].

The mechanism by which OV kills tumours includes infection and replication in tumour cells, leading to the lysis or apoptosis of the tumour cells, and a more important mechanism is the killed tumour cells release tumour-related antigens to achieve an enhanced tumour-specific immune response [34]. Since OV therapy can induce an adaptive antitumour immune response, a combination of immune checkpoint inhibitors (ICIs) and OVs could have potential clinical value. Some studies have shown that OV combined with CTLA-4 inhibitors or PD-1 inhibitors improves the antitumour response and significantly improves patient survival, both in preclinical studies and clinical trials [2, 35–38]. Currently, there are 19 ongoing clinical trials of ICI and OV combination therapy, showing that combination therapy has become one of the hot spots of OV clinical treatment options [39].

However, the existing combination therapy focuses more on the adaptive immune response and less on innate immunity. When OVs lyse tumour cells, they release many cytokines, pathogen-associated molecular patterns (PAMPs) and damage-associated molecular patterns (DAMPs), which activate innate immune responses. Our data showed that the lysate of OH2 after infection with tumour cells could effectively stimulate mouse macrophage line RAW264.7 activation and M1-like polarization in vitro. Although RNA sequencing results showed that RAW264.7 cells had a strong antiviral immune response, M1-like polarized RAW264.7 cells also showed significant tumour-specific killing ability in subsequent experiments. Saha et al. [40] showed that ICI combined with OV modestly extended the survival of a mouse glioma model, which was associated with macrophage influx and M1-like polarization. These results suggest that OV combined with innate immune cells, such as macrophages and dendritic cells, also has potential application value.

Recent studies have shown that the CD47-SIRPα axis is a phagocytic checkpoint regulating macrophages and other innate immune cells, and a series of molecules that block the CD47-SIRPα axis are in clinical development for tumour therapy [13, 41]. Some research groups have engineered oHSV1 expressing a full-length CD47 antibody, which has achieved good therapeutic efficacy in mouse models of metastatic ovarian cancer and glioblastoma [42, 43]. Yao et al. [44] reported the antitumour effect of a novel oncolytic adenovirus containing the SIRPα-IgG1 Fc fusion gene in CD47-positive cancer. Since CD47 is commonly expressed at high levels in normal tissues, specific targeting of tumour cells via CD47 is questionable [45]. SIRPα is highly expressed in bone marrow cells such as macrophages and DCs but is expressed at low levels in other immune cells. Therefore, SIRPα may be a more ideal target for the CD47-SIRPα axis [45, 46]. In this study, we demonstrated that oncolytic virus therapy could effectively recruit and activate macrophages in vivo and induce M1-like polarization, demonstrating the feasibility of blocking OVs in combination with blocking the CD47-SIRPα axis. The combination of OH2 and anti-SIRPα antibodies had a significant effect on the treatment of the mouse colorectal cancer CT-26 model. Interestingly, we found that the combination therapy was more effective in mice with larger initial tumour volumes. This may be due to the number of infiltrated monocytes in the tumour microenvironment with increasing tumour size, and oncolytic virus treatment switched the tumour microenvironment from “cold” to “hot”, thus inducing the generation of more abundant M1-like macrophages. These results also suggest that the therapeutic effect can be further improved by optimizing different dosing times of combination therapy.

With better understanding of the tumour immune microenvironment, immunotherapy based on macrophages is becoming a reality [47]. Macrophages are important regulators of many aspects of the TME and can activate tumour-specific immune responses directly or through nonspecific effects on T and B-cell functions [12, 48, 49]. We also observed that either OV therapy alone or in combination with SIRPα antibody recruited large numbers of NK cells and monocytes to trigger innate immune responses, which is consistent with the findings of Ramelyte et al. [50]. Macrophages play an important role in limiting HSV infection [51, 52], and the single-cell sequencing results showed activation of TLR signalling pathways involved in the combination therapy group. We observed that the combination therapy did not produce a significant antiviral response compared with treatment alone, however, macrophages are more likely to activate the overall immune state. The results suggest that the combination therapy induces a more specific killing of tumour cells. The characteristics of combination therapy at the cellular and molecular levels are based on the rapid reconstruction of the immune environment within the TME and the comprehensive and continuous activation of innate and adaptive immunity. The effectiveness of combination therapy is due to multiple immune synergies, not just one type of immune cell. Macrophages may play a role in early antigen presentation and an expanded immune response.

## Conclusions

In conclusion, our data support the feasibility of oncolytic virus therapy in combination with an anti-SIRPα antibody. The introduction of the anti-SIRPα antibody can accelerate cellular reconstitution of the TME and induce a specific antitumour immune response through an earlier innate immune response. Our study suggests a new strategy for oncolytic virus therapy.

## Supplementary Information

**Additional file 1: Table S1.** Gene lis for heatmap.

**Additional file 2: Table S2.** GSVA gene list.

**Additional file 3: Figure S1.** Cell proliferation assay results. Cell proliferation assay results of Raw264.7 cell lines treated with OH2 MOI=1 (red), OH2 MOI=0.5 (blue) and untreated (black) group by CCK8 assay in 72 hours. **Figure S2.** OH2 lysates induce RAW264.7 polarization and phagocytosis in vitro. A. Demonstration of the analysis of the phagocytosis by flow cytometry. B. Cell proliferation assay results of CT26, MC38 and 4T-1 cell lines treated with lysate (red), CFS (blue), Cell frozen lysate (purple) and untreated (black) group by CCK8 assay in 24 hours. **, *p*<0.01. **Figure S3.** The ratio of M1 (F4/80+CD86+) and M2 (F4/80+CD206+) macrophages in RAW264.7 without any treatment by flow cytometry. **Figure S4.** The ratio of M1 (F4/80+CD86+) and M2 (F4/80+CD206+) macrophages in RAW264.7 treated with lysate in the blocked SIRPα group and the non-blocked SIRPα group. A. Demonstration of the analysis of the polarization of macrophages by flow cytometry. B. Display of isotype control results for different antibodies. C. The ratio of M1 (F4/80+CD86+) and M2 (F4/80+CD206+) macrophages in the blocked SIRPα group and the non-blocked SIRPα group. D. The ratio of M1 (F4/80+CD86+) and M2 (F4/80+CD206+) macrophages in the blocked SIRPα group and the non-blocked SIRPα group. An unpaired Student’s t test was used to analyze the significance of the difference between two groups. **Figure S5.** Cell frozen lysate and CFS induce RAW264.7 polarization in vitro. A. Flow cytometric analysis results of one representative sample from each cell line. B. The ratio of M1 (F4/80+CD86+) subtype in the Cell frozen lysate and CFS groups of CT26, MC38 and 4T-1 cells detected by flow cytometry. An unpaired Student’s t test was used to analyze the significance of the difference between two groups. **Figure S6.** OH2 lysates induce primary macrophages polarization in vitro. A. Demonstration of the analysis of the polarization of macrophages by flow cytometry. B. The percentage of M1 (F4/80+CD86+) subtype and M2 (F4/80+CD206+) subtype in the lysate, CFS, untreated and cell frozen lysate groups detected by flow cytometry. The data are averages from three samples per treatment group. Statistical analysis was performed using ANOVA with multiple comparisons. ns, no significant differences, *, *p*<0.05, **, *p*<0.01, ***, *p*<0.001, ****, *p*<0.0001. **Figure S7.** The clear efficiency of CL detected by flow cytometry. Statistical analysis was performed using ANOVA with multiple comparisons. ***, *p*=0.0003; ****, *p*<0.0001. **Figure S8.** Tumor-rechallenge with CT26 of the OH2 or OH2+ CL cured animals. A. The tumor growth curve of CT26 cells rechallenge after the tumors of the CL model had completely regressed. B. The tumorigenic rate of tumor-rechallenge mice in OH2 combined control liposomes group and OH2 combined CL group. Statistical differences were calculated using the Chi-square test. *p*=0.0291. **Figure S9.** M-IHC staning of OH2 (left) and OH2 combined CL group (right) including each single staining marker. **Figure S10.** Expression level of SIRPα on macrophages in spleen from mice detected by flow cytometry. A. Demonstration of the expression level of SIRPα on macrophages in spleen from mice detected by flow cytometry. B. Histogram showing Expression level of SIRPα on macrophages in spleen from mice (*n* = 3). **Figure S11.** Combined anti-SIRPα therapy with OH2 enhances anticancer immune responses in a CT26 cancer model. A. The treatment process and experimental timeline for the combined treatment. B. The tumor growth curve of different treatment groups of tumor-bearing mice with smaller tumor volumes at the initial treatment. The red box zoomed in to show the OH2 combined anti-SIRPα antibody group, OH2 combined isotype and OH2 group. Two-way analysis of variance (ANOVA) was performed on the experimental data. *n*=10 mice/group. ns, no significant differences. C. The survival curve of different treatment groups of tumor-bearing mice with smaller tumor volumes at the initial treatment. The Kaplan-Meier method and log-rank test were used for the survival curve data. ns, no significant differences. **Figure S12.** M-IHC staining results of anti-SIRPα model. M-IHC stanning of control group (top left), OH2 group (top right), OH2 combined isotype group (bottom left) and OH2 combined anti-SIRPα antibody group (bottom right) including each single staining marker. **Figure S13.** IHC staining and quantitative results of the anti-SIRPα antibody treatment model. A. Representative IHC staining for CD8, F4/80, CD86 and CD206 of the OH2 combined anti-SIRPα antibody group, control group and anti-SIRPα antibody group with larger tumor volumes at the initial treatment. B. Quantitative results of CD8, F4/80, CD86 and CD206 staining in the the OH2 combined anti-SIRPα antibody group, control group and anti-SIRPα antibody group. Original magnification, ×200. *n*=5 samples/group. Statistical analysis was performed using ANOVA with multiple comparisons. ns, no significant differences, *, *p*<0.05, **, *p*<0.01, ***, *p*<0.001, ****, *p*<0.0001. **Figure S14.** KEGG results of tumor tissues from anti-SIRPα model. A. KEGG results of differentially expressed genes between OH2 combined anti-SIRPα antibody group and control group. B. KEGG results of differentially expressed genes between OH2 combined anti-SIRPα antibody group and OH2 combined Isotype antibody group. **Figure S15.** GSEA results of tumor tissues from anti-SIRPα model. A. GSEA results of differentially expressed genes between OH2 combined anti-SIRPα antibody group and control group. B. GSEA results of differentially expressed genes between OH2 combined anti-SIRPα antibody group and oHSV-2 combined Isotype antibody group. **Figure S16.** scRNA-seq data analysis. A. UMAP plots represent the clusters (left) and groups (right) of tumor cells. B. A heatmap showing the scaled average expression of each cell subcluster. C. UMAP plots showing the expression values of canonical marker genes of each subcluster. D. A heatmap showing the scaled average expression of each immune cell subcluster. C. UMAP plots showing the expression values of canonical marker genes of each immune subcluster. **Figure S17.** CTL Assay. A. The killing functions of lymphocyte co-cultured with different cancer cell lysates detected by CFSE/PI. Three effectors to target ratios were set for each cell line (Lymphocyte: tumour cells=25:1, 50:1, and 100:1). The data are averages from three samples per treatment group. Statistical analysis was performed using ANOVA with multiple comparisons. ns, no significant differences, *, *p*<0.05, **, *p*<0.01, ***, *p*<0.001, ****, *p*<0.0001. B. Supernatant from the CTL assay was tested for TNF-α, Granzyme B and IFN-γ by ELISA. Statistical analysis was performed using ANOVA with multiple comparisons. ns, no significant differences, ****, *p*<0.0001.

**Additional file 4.** Supplementary methods.

## Data Availability

The data associated with this study are present in the paper or the Supplementary Materials. The RNA-seq data is also available from the corresponding authors upon reasonable request.

## Abbreviations

SIRPα: Signal regulatory protein-alpha
OH2: Oncolytic herpes simplex virus 2
OV: Oncolytic virus
RNA-seq: RNA sequencing
TME: Tumour microenvironment environment
ICB: Immune checkpoint blockade
ICD: Immunogenic cell death
DAMPs: Damage-associated molecular patterns
PAMPs: Pathogen-associated molecular patterns
CFS: Cell-free supernatant
CCK8: Cell Counting Kit-8
GO: Gene ontology
KEGG: Kyoto Encyclopedia of Genes and Genomes
GSEA: Gene set enrichment analysis
CL: Clodronate liposomes
M-IHC: Multicolour immunohistochemical
IHC: Immunohistochemical
SPF: Specific pathogen free
TLR: Toll-like receptor
CTL: Cytotoxic T lymphocyte
