# Supplementary figures and images for: SIRPα antibody combined with oncolytic virus OH2 protects against tumours by activating innate immunity and reprogramming the tumour immune microenvironment
Source: BMC Med. 2022 Oct 31;20:376. doi: 10.1186/s12916-022-02574-z (PMC9620659; doi:10.1186/s12916-022-02574-z)

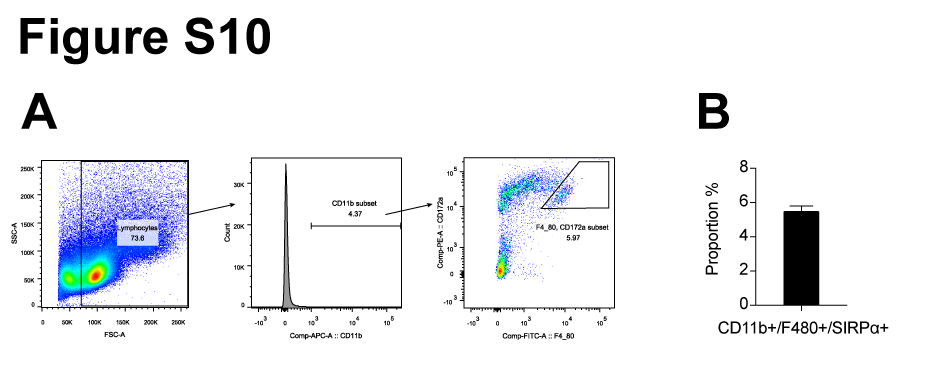

Supplement: Supplementary file 3 — Additional file 3: Figure S1. Cell proliferation assay results. Cell proliferation assay results of Raw264.7 cell lines treated with OH2 MOI=1 (red), OH2 MOI=0.5 (blue) and untreated (black) group by CCK8 assay in 72 hours. Figure S2. OH2 lysates induce RAW264.7 polarization and phagocytosis in vitro. A. Demonstration of the analysis of the phagocytosis by flow cytometry. B. Cell proliferation assay results of CT26, MC38 and 4T-1 cell lines treated with lysate (red), CFS (blue), Cell frozen lysate (purple) and untreated (black) group by CCK8 assay in 24 hours. **, p<0.01. Figure S3. The ratio of M1 (F4/80+CD86+) and M2 (F4/80+CD206+) macrophages in RAW264.7 without any treatment by flow cytometry. Figure S4. The ratio of M1 (F4/80+CD86+) and M2 (F4/80+CD206+) macrophages in RAW264.7 treated with lysate in the blocked SIRPα group and the non-blocked SIRPα group. A. Demonstration of the analysis of the polarization of macrophages by flow cytometry. B. Display of isotype control results for different antibodies. C. The ratio of M1 (F4/80+CD86+) and M2 (F4/80+CD206+) macrophages in the blocked SIRPα group and the non-blocked SIRPα group. D. The ratio of M1 (F4/80+CD86+) and M2 (F4/80+CD206+) macrophages in the blocked SIRPα group and the non-blocked SIRPα group. An unpaired Student’s t test was used to analyze the significance of the difference between two groups. Figure S5. Cell frozen lysate and CFS induce RAW264.7 polarization in vitro. A. Flow cytometric analysis results of one representative sample from each cell line. B. The ratio of M1 (F4/80+CD86+) subtype in the Cell frozen lysate and CFS groups of CT26, MC38 and 4T-1 cells detected by flow cytometry. An unpaired Student’s t test was used to analyze the significance of the difference between two groups. Figure S6. OH2 lysates induce primary macrophages polarization in vitro. A. Demonstration of the analysis of the polarization of macrophages by flow cytometry. B. The percentage of M1 (F4/80+CD86+) subtype and M [file 12916_2022_2574_MOESM3_ESM.zip › Additional file 3 Figure S10R3.tif]

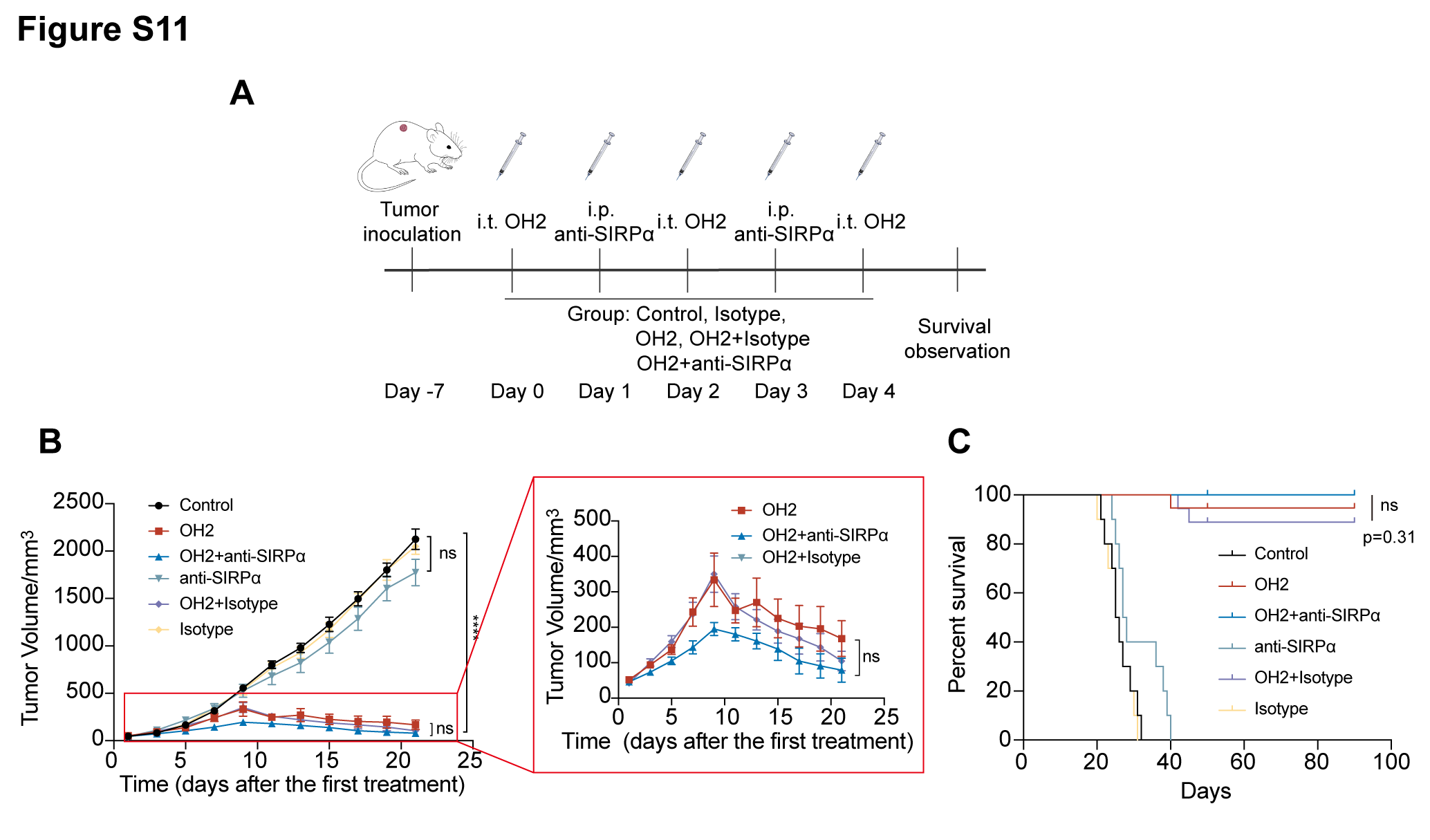

Supplement: Supplementary file 3 — Additional file 3: Figure S1. Cell proliferation assay results. Cell proliferation assay results of Raw264.7 cell lines treated with OH2 MOI=1 (red), OH2 MOI=0.5 (blue) and untreated (black) group by CCK8 assay in 72 hours. Figure S2. OH2 lysates induce RAW264.7 polarization and phagocytosis in vitro. A. Demonstration of the analysis of the phagocytosis by flow cytometry. B. Cell proliferation assay results of CT26, MC38 and 4T-1 cell lines treated with lysate (red), CFS (blue), Cell frozen lysate (purple) and untreated (black) group by CCK8 assay in 24 hours. **, p<0.01. Figure S3. The ratio of M1 (F4/80+CD86+) and M2 (F4/80+CD206+) macrophages in RAW264.7 without any treatment by flow cytometry. Figure S4. The ratio of M1 (F4/80+CD86+) and M2 (F4/80+CD206+) macrophages in RAW264.7 treated with lysate in the blocked SIRPα group and the non-blocked SIRPα group. A. Demonstration of the analysis of the polarization of macrophages by flow cytometry. B. Display of isotype control results for different antibodies. C. The ratio of M1 (F4/80+CD86+) and M2 (F4/80+CD206+) macrophages in the blocked SIRPα group and the non-blocked SIRPα group. D. The ratio of M1 (F4/80+CD86+) and M2 (F4/80+CD206+) macrophages in the blocked SIRPα group and the non-blocked SIRPα group. An unpaired Student’s t test was used to analyze the significance of the difference between two groups. Figure S5. Cell frozen lysate and CFS induce RAW264.7 polarization in vitro. A. Flow cytometric analysis results of one representative sample from each cell line. B. The ratio of M1 (F4/80+CD86+) subtype in the Cell frozen lysate and CFS groups of CT26, MC38 and 4T-1 cells detected by flow cytometry. An unpaired Student’s t test was used to analyze the significance of the difference between two groups. Figure S6. OH2 lysates induce primary macrophages polarization in vitro. A. Demonstration of the analysis of the polarization of macrophages by flow cytometry. B. The percentage of M1 (F4/80+CD86+) subtype and M [file 12916_2022_2574_MOESM3_ESM.zip › Additional file 3 Figure S11R3.tif]

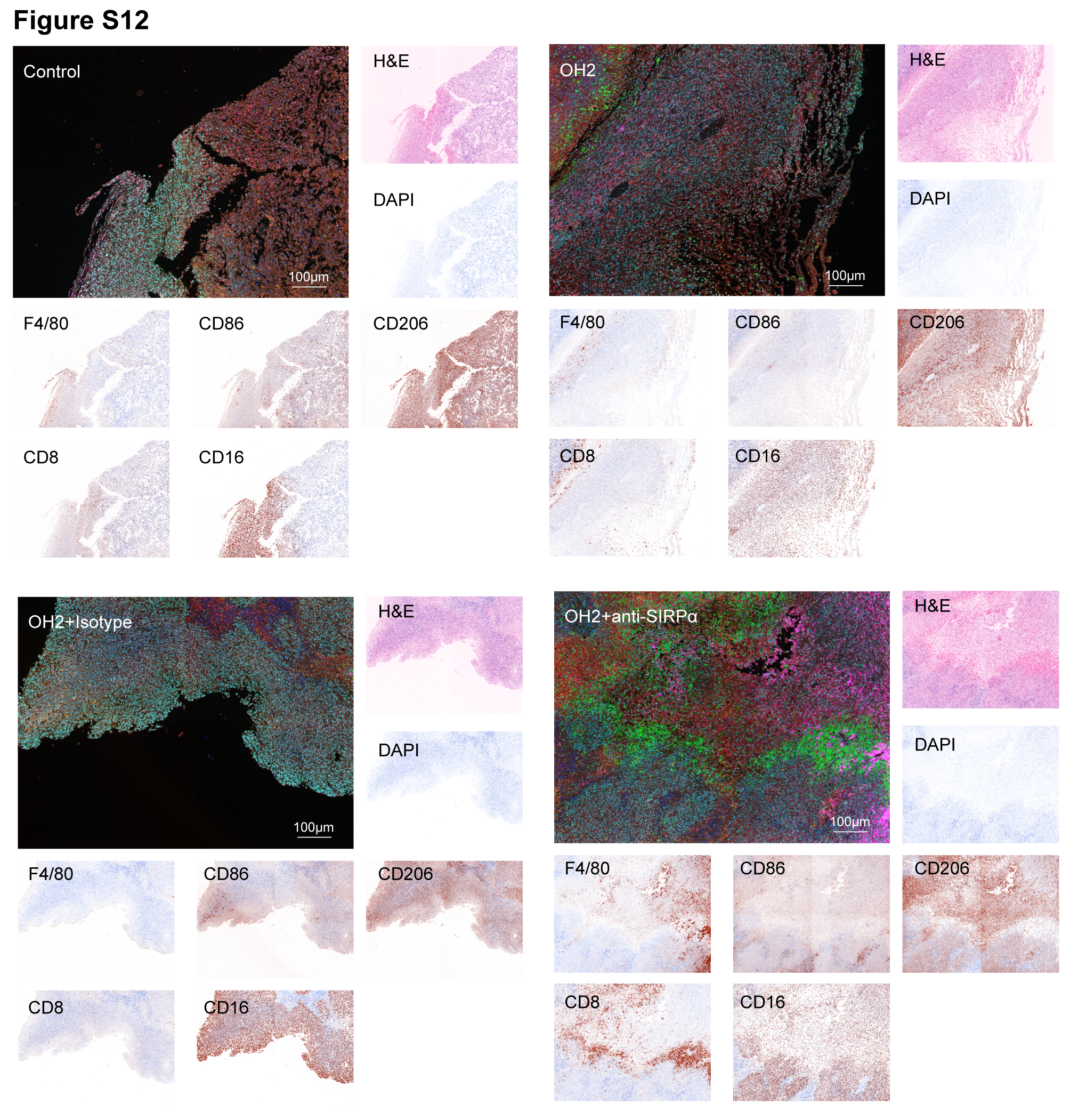

Supplement: Supplementary file 3 — Additional file 3: Figure S1. Cell proliferation assay results. Cell proliferation assay results of Raw264.7 cell lines treated with OH2 MOI=1 (red), OH2 MOI=0.5 (blue) and untreated (black) group by CCK8 assay in 72 hours. Figure S2. OH2 lysates induce RAW264.7 polarization and phagocytosis in vitro. A. Demonstration of the analysis of the phagocytosis by flow cytometry. B. Cell proliferation assay results of CT26, MC38 and 4T-1 cell lines treated with lysate (red), CFS (blue), Cell frozen lysate (purple) and untreated (black) group by CCK8 assay in 24 hours. **, p<0.01. Figure S3. The ratio of M1 (F4/80+CD86+) and M2 (F4/80+CD206+) macrophages in RAW264.7 without any treatment by flow cytometry. Figure S4. The ratio of M1 (F4/80+CD86+) and M2 (F4/80+CD206+) macrophages in RAW264.7 treated with lysate in the blocked SIRPα group and the non-blocked SIRPα group. A. Demonstration of the analysis of the polarization of macrophages by flow cytometry. B. Display of isotype control results for different antibodies. C. The ratio of M1 (F4/80+CD86+) and M2 (F4/80+CD206+) macrophages in the blocked SIRPα group and the non-blocked SIRPα group. D. The ratio of M1 (F4/80+CD86+) and M2 (F4/80+CD206+) macrophages in the blocked SIRPα group and the non-blocked SIRPα group. An unpaired Student’s t test was used to analyze the significance of the difference between two groups. Figure S5. Cell frozen lysate and CFS induce RAW264.7 polarization in vitro. A. Flow cytometric analysis results of one representative sample from each cell line. B. The ratio of M1 (F4/80+CD86+) subtype in the Cell frozen lysate and CFS groups of CT26, MC38 and 4T-1 cells detected by flow cytometry. An unpaired Student’s t test was used to analyze the significance of the difference between two groups. Figure S6. OH2 lysates induce primary macrophages polarization in vitro. A. Demonstration of the analysis of the polarization of macrophages by flow cytometry. B. The percentage of M1 (F4/80+CD86+) subtype and M [file 12916_2022_2574_MOESM3_ESM.zip › Additional file 3 Figure S12R3.tif]

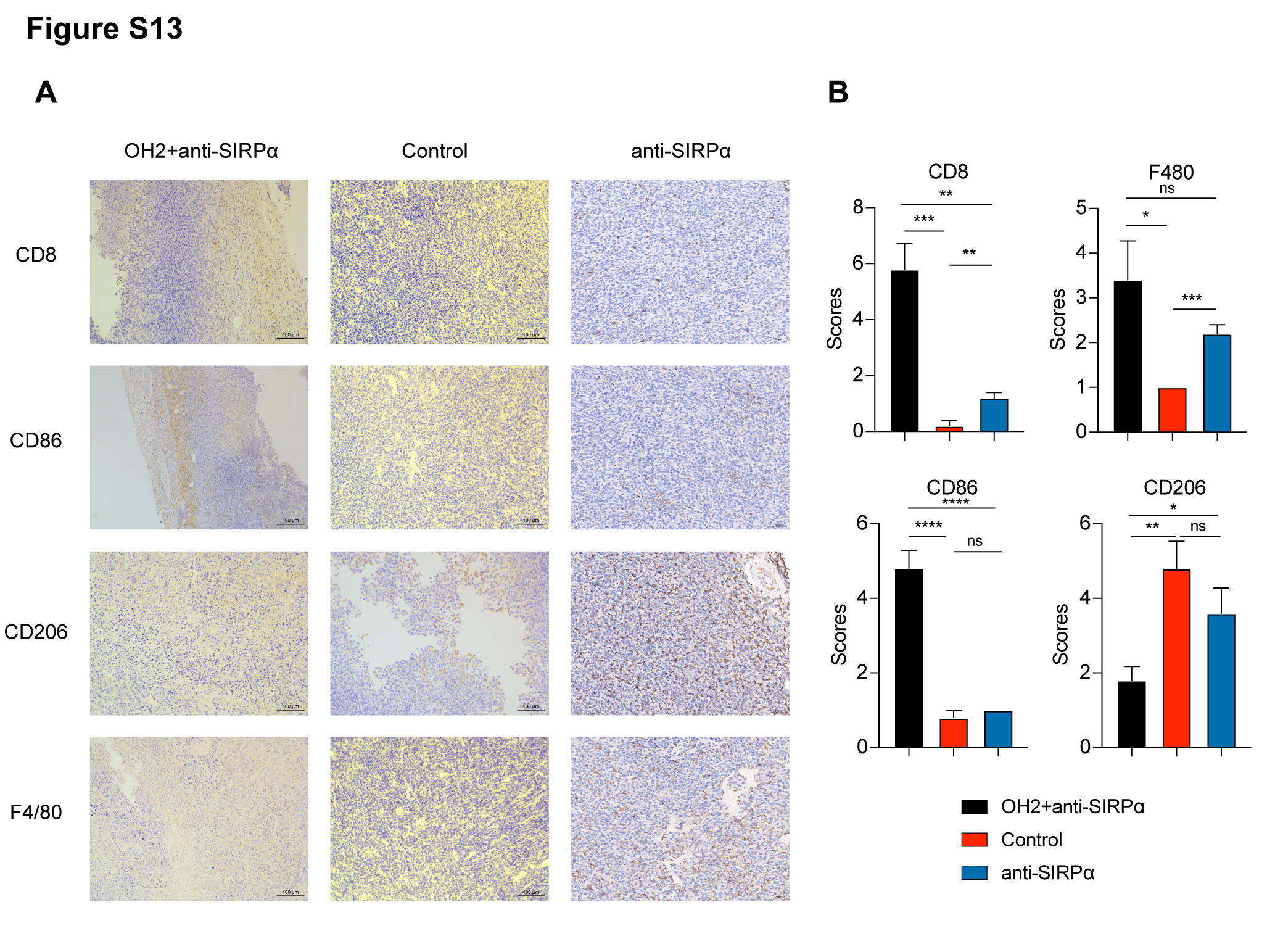

Supplement: Supplementary file 3 — Additional file 3: Figure S1. Cell proliferation assay results. Cell proliferation assay results of Raw264.7 cell lines treated with OH2 MOI=1 (red), OH2 MOI=0.5 (blue) and untreated (black) group by CCK8 assay in 72 hours. Figure S2. OH2 lysates induce RAW264.7 polarization and phagocytosis in vitro. A. Demonstration of the analysis of the phagocytosis by flow cytometry. B. Cell proliferation assay results of CT26, MC38 and 4T-1 cell lines treated with lysate (red), CFS (blue), Cell frozen lysate (purple) and untreated (black) group by CCK8 assay in 24 hours. **, p<0.01. Figure S3. The ratio of M1 (F4/80+CD86+) and M2 (F4/80+CD206+) macrophages in RAW264.7 without any treatment by flow cytometry. Figure S4. The ratio of M1 (F4/80+CD86+) and M2 (F4/80+CD206+) macrophages in RAW264.7 treated with lysate in the blocked SIRPα group and the non-blocked SIRPα group. A. Demonstration of the analysis of the polarization of macrophages by flow cytometry. B. Display of isotype control results for different antibodies. C. The ratio of M1 (F4/80+CD86+) and M2 (F4/80+CD206+) macrophages in the blocked SIRPα group and the non-blocked SIRPα group. D. The ratio of M1 (F4/80+CD86+) and M2 (F4/80+CD206+) macrophages in the blocked SIRPα group and the non-blocked SIRPα group. An unpaired Student’s t test was used to analyze the significance of the difference between two groups. Figure S5. Cell frozen lysate and CFS induce RAW264.7 polarization in vitro. A. Flow cytometric analysis results of one representative sample from each cell line. B. The ratio of M1 (F4/80+CD86+) subtype in the Cell frozen lysate and CFS groups of CT26, MC38 and 4T-1 cells detected by flow cytometry. An unpaired Student’s t test was used to analyze the significance of the difference between two groups. Figure S6. OH2 lysates induce primary macrophages polarization in vitro. A. Demonstration of the analysis of the polarization of macrophages by flow cytometry. B. The percentage of M1 (F4/80+CD86+) subtype and M [file 12916_2022_2574_MOESM3_ESM.zip › Additional file 3 Figure S13R3.tif]

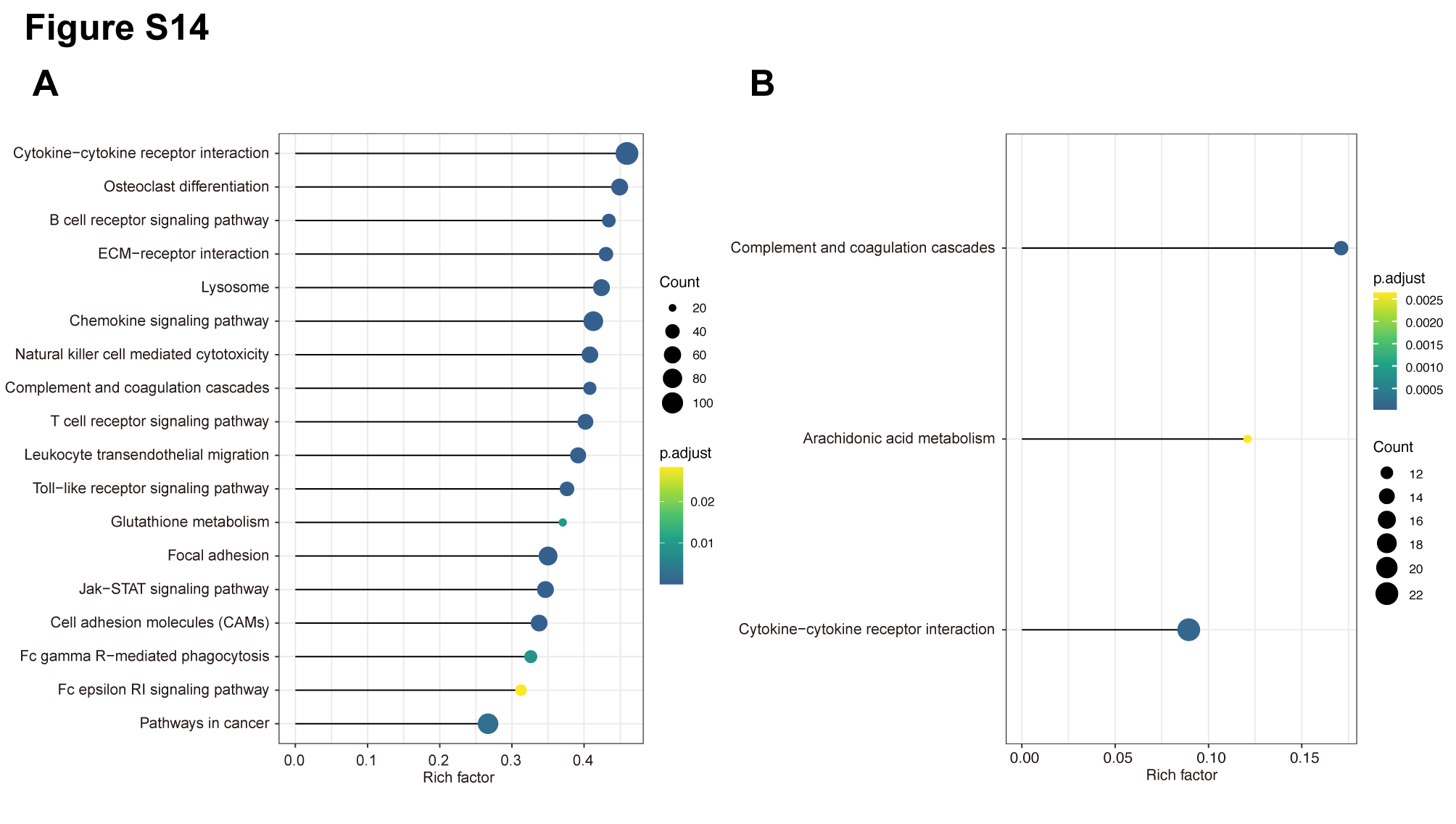

Supplement: Supplementary file 3 — Additional file 3: Figure S1. Cell proliferation assay results. Cell proliferation assay results of Raw264.7 cell lines treated with OH2 MOI=1 (red), OH2 MOI=0.5 (blue) and untreated (black) group by CCK8 assay in 72 hours. Figure S2. OH2 lysates induce RAW264.7 polarization and phagocytosis in vitro. A. Demonstration of the analysis of the phagocytosis by flow cytometry. B. Cell proliferation assay results of CT26, MC38 and 4T-1 cell lines treated with lysate (red), CFS (blue), Cell frozen lysate (purple) and untreated (black) group by CCK8 assay in 24 hours. **, p<0.01. Figure S3. The ratio of M1 (F4/80+CD86+) and M2 (F4/80+CD206+) macrophages in RAW264.7 without any treatment by flow cytometry. Figure S4. The ratio of M1 (F4/80+CD86+) and M2 (F4/80+CD206+) macrophages in RAW264.7 treated with lysate in the blocked SIRPα group and the non-blocked SIRPα group. A. Demonstration of the analysis of the polarization of macrophages by flow cytometry. B. Display of isotype control results for different antibodies. C. The ratio of M1 (F4/80+CD86+) and M2 (F4/80+CD206+) macrophages in the blocked SIRPα group and the non-blocked SIRPα group. D. The ratio of M1 (F4/80+CD86+) and M2 (F4/80+CD206+) macrophages in the blocked SIRPα group and the non-blocked SIRPα group. An unpaired Student’s t test was used to analyze the significance of the difference between two groups. Figure S5. Cell frozen lysate and CFS induce RAW264.7 polarization in vitro. A. Flow cytometric analysis results of one representative sample from each cell line. B. The ratio of M1 (F4/80+CD86+) subtype in the Cell frozen lysate and CFS groups of CT26, MC38 and 4T-1 cells detected by flow cytometry. An unpaired Student’s t test was used to analyze the significance of the difference between two groups. Figure S6. OH2 lysates induce primary macrophages polarization in vitro. A. Demonstration of the analysis of the polarization of macrophages by flow cytometry. B. The percentage of M1 (F4/80+CD86+) subtype and M [file 12916_2022_2574_MOESM3_ESM.zip › Additional file 3 Figure S14R3.tif]

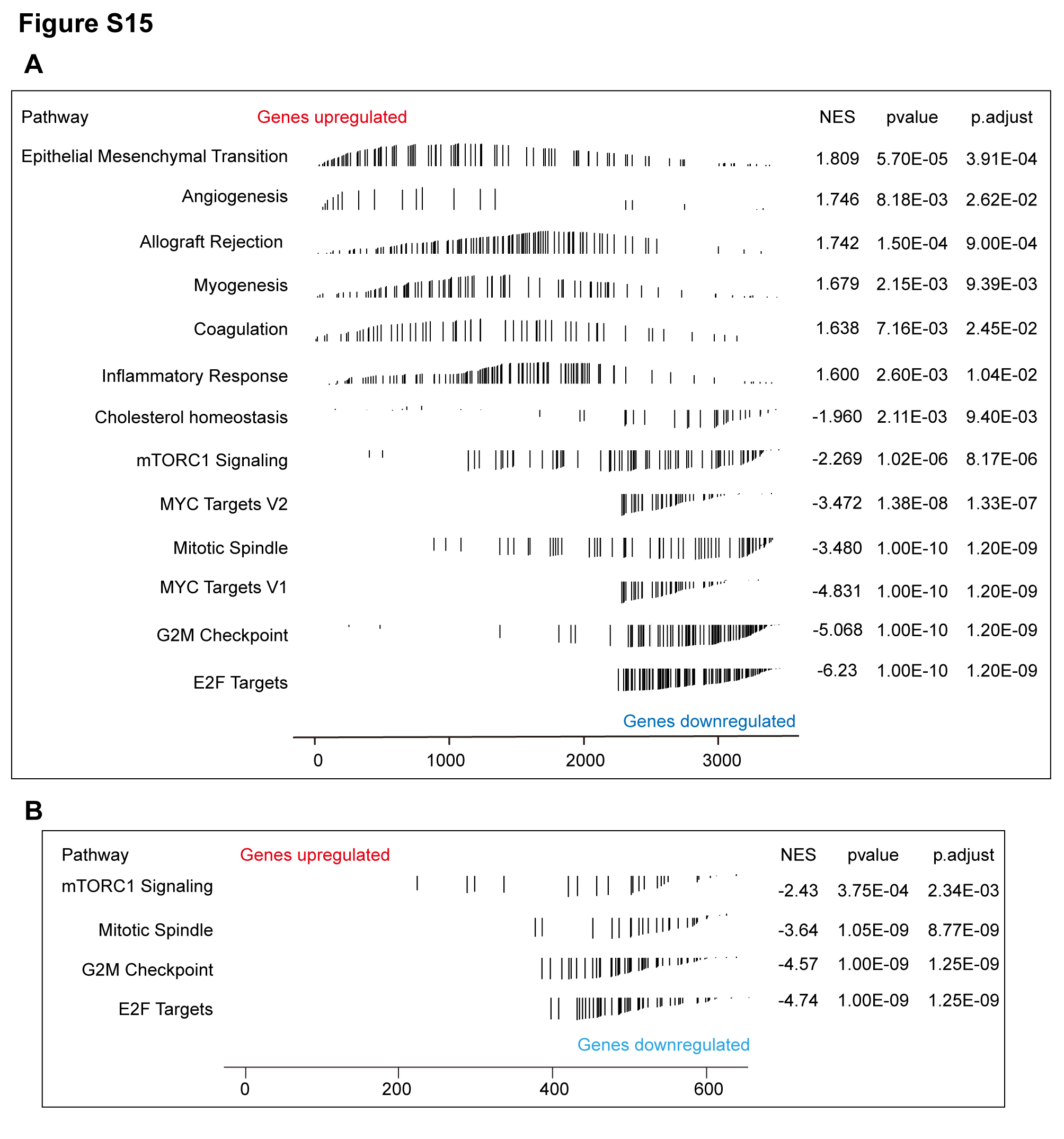

Supplement: Supplementary file 3 — Additional file 3: Figure S1. Cell proliferation assay results. Cell proliferation assay results of Raw264.7 cell lines treated with OH2 MOI=1 (red), OH2 MOI=0.5 (blue) and untreated (black) group by CCK8 assay in 72 hours. Figure S2. OH2 lysates induce RAW264.7 polarization and phagocytosis in vitro. A. Demonstration of the analysis of the phagocytosis by flow cytometry. B. Cell proliferation assay results of CT26, MC38 and 4T-1 cell lines treated with lysate (red), CFS (blue), Cell frozen lysate (purple) and untreated (black) group by CCK8 assay in 24 hours. **, p<0.01. Figure S3. The ratio of M1 (F4/80+CD86+) and M2 (F4/80+CD206+) macrophages in RAW264.7 without any treatment by flow cytometry. Figure S4. The ratio of M1 (F4/80+CD86+) and M2 (F4/80+CD206+) macrophages in RAW264.7 treated with lysate in the blocked SIRPα group and the non-blocked SIRPα group. A. Demonstration of the analysis of the polarization of macrophages by flow cytometry. B. Display of isotype control results for different antibodies. C. The ratio of M1 (F4/80+CD86+) and M2 (F4/80+CD206+) macrophages in the blocked SIRPα group and the non-blocked SIRPα group. D. The ratio of M1 (F4/80+CD86+) and M2 (F4/80+CD206+) macrophages in the blocked SIRPα group and the non-blocked SIRPα group. An unpaired Student’s t test was used to analyze the significance of the difference between two groups. Figure S5. Cell frozen lysate and CFS induce RAW264.7 polarization in vitro. A. Flow cytometric analysis results of one representative sample from each cell line. B. The ratio of M1 (F4/80+CD86+) subtype in the Cell frozen lysate and CFS groups of CT26, MC38 and 4T-1 cells detected by flow cytometry. An unpaired Student’s t test was used to analyze the significance of the difference between two groups. Figure S6. OH2 lysates induce primary macrophages polarization in vitro. A. Demonstration of the analysis of the polarization of macrophages by flow cytometry. B. The percentage of M1 (F4/80+CD86+) subtype and M [file 12916_2022_2574_MOESM3_ESM.zip › Additional file 3 Figure S15R3.tif]

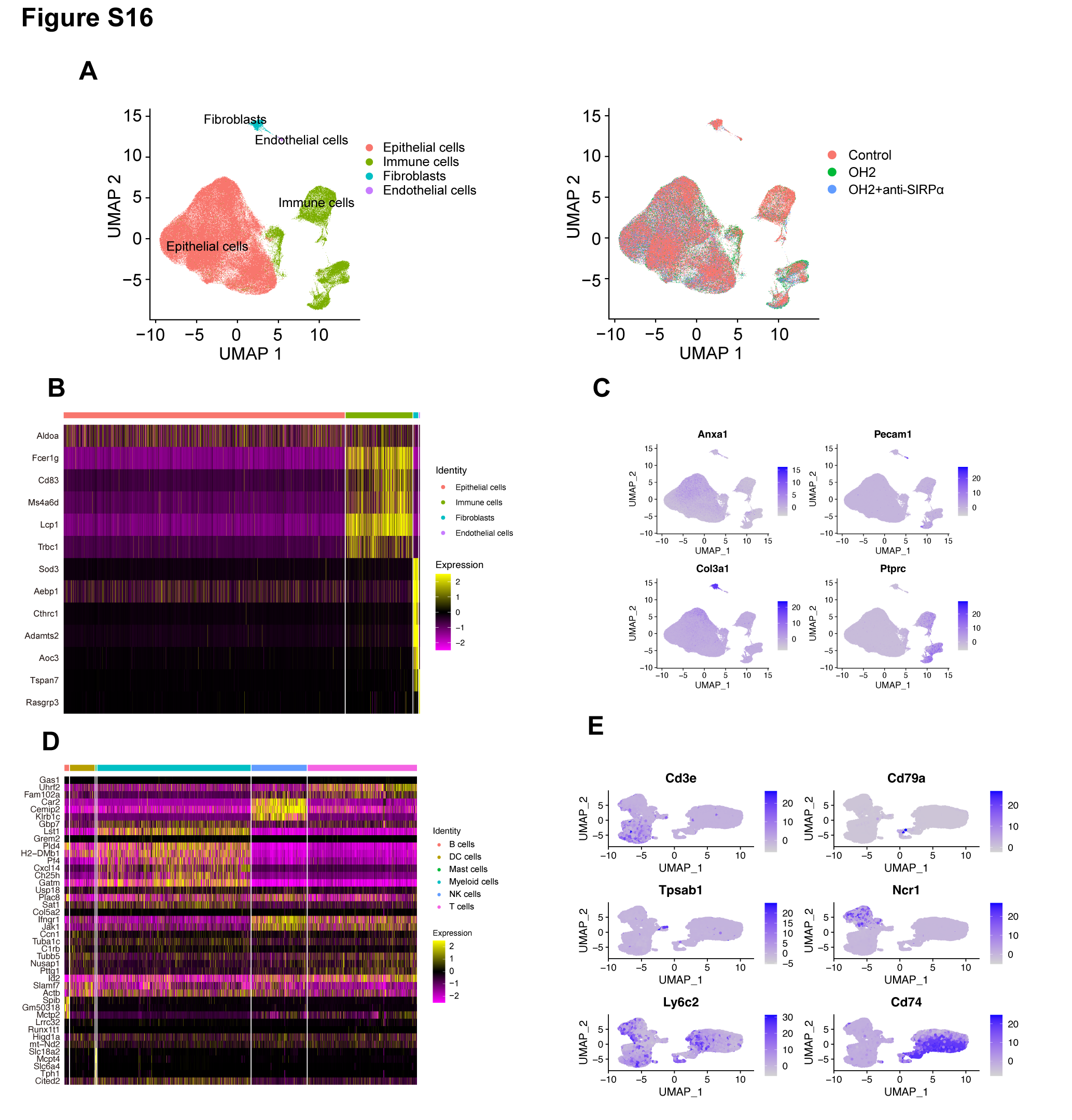

Supplement: Supplementary file 3 — Additional file 3: Figure S1. Cell proliferation assay results. Cell proliferation assay results of Raw264.7 cell lines treated with OH2 MOI=1 (red), OH2 MOI=0.5 (blue) and untreated (black) group by CCK8 assay in 72 hours. Figure S2. OH2 lysates induce RAW264.7 polarization and phagocytosis in vitro. A. Demonstration of the analysis of the phagocytosis by flow cytometry. B. Cell proliferation assay results of CT26, MC38 and 4T-1 cell lines treated with lysate (red), CFS (blue), Cell frozen lysate (purple) and untreated (black) group by CCK8 assay in 24 hours. **, p<0.01. Figure S3. The ratio of M1 (F4/80+CD86+) and M2 (F4/80+CD206+) macrophages in RAW264.7 without any treatment by flow cytometry. Figure S4. The ratio of M1 (F4/80+CD86+) and M2 (F4/80+CD206+) macrophages in RAW264.7 treated with lysate in the blocked SIRPα group and the non-blocked SIRPα group. A. Demonstration of the analysis of the polarization of macrophages by flow cytometry. B. Display of isotype control results for different antibodies. C. The ratio of M1 (F4/80+CD86+) and M2 (F4/80+CD206+) macrophages in the blocked SIRPα group and the non-blocked SIRPα group. D. The ratio of M1 (F4/80+CD86+) and M2 (F4/80+CD206+) macrophages in the blocked SIRPα group and the non-blocked SIRPα group. An unpaired Student’s t test was used to analyze the significance of the difference between two groups. Figure S5. Cell frozen lysate and CFS induce RAW264.7 polarization in vitro. A. Flow cytometric analysis results of one representative sample from each cell line. B. The ratio of M1 (F4/80+CD86+) subtype in the Cell frozen lysate and CFS groups of CT26, MC38 and 4T-1 cells detected by flow cytometry. An unpaired Student’s t test was used to analyze the significance of the difference between two groups. Figure S6. OH2 lysates induce primary macrophages polarization in vitro. A. Demonstration of the analysis of the polarization of macrophages by flow cytometry. B. The percentage of M1 (F4/80+CD86+) subtype and M [file 12916_2022_2574_MOESM3_ESM.zip › Additional file 3 Figure S16R3.tif]

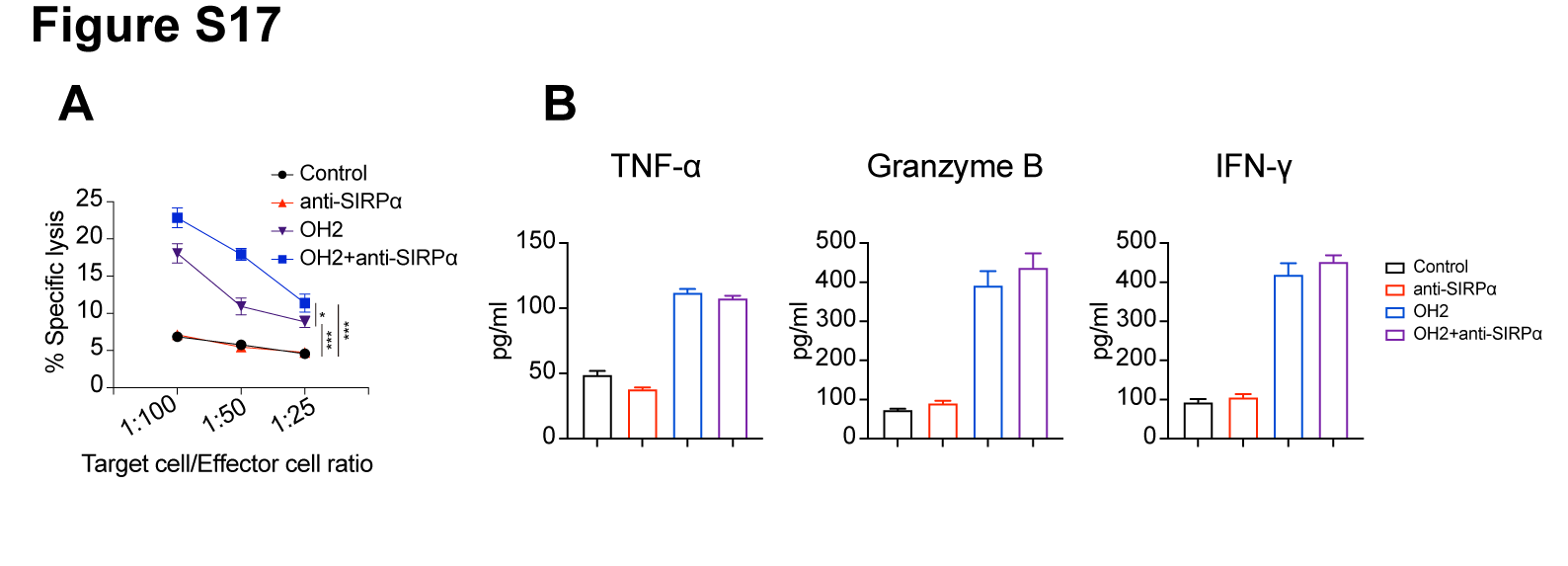

Supplement: Supplementary file 3 — Additional file 3: Figure S1. Cell proliferation assay results. Cell proliferation assay results of Raw264.7 cell lines treated with OH2 MOI=1 (red), OH2 MOI=0.5 (blue) and untreated (black) group by CCK8 assay in 72 hours. Figure S2. OH2 lysates induce RAW264.7 polarization and phagocytosis in vitro. A. Demonstration of the analysis of the phagocytosis by flow cytometry. B. Cell proliferation assay results of CT26, MC38 and 4T-1 cell lines treated with lysate (red), CFS (blue), Cell frozen lysate (purple) and untreated (black) group by CCK8 assay in 24 hours. **, p<0.01. Figure S3. The ratio of M1 (F4/80+CD86+) and M2 (F4/80+CD206+) macrophages in RAW264.7 without any treatment by flow cytometry. Figure S4. The ratio of M1 (F4/80+CD86+) and M2 (F4/80+CD206+) macrophages in RAW264.7 treated with lysate in the blocked SIRPα group and the non-blocked SIRPα group. A. Demonstration of the analysis of the polarization of macrophages by flow cytometry. B. Display of isotype control results for different antibodies. C. The ratio of M1 (F4/80+CD86+) and M2 (F4/80+CD206+) macrophages in the blocked SIRPα group and the non-blocked SIRPα group. D. The ratio of M1 (F4/80+CD86+) and M2 (F4/80+CD206+) macrophages in the blocked SIRPα group and the non-blocked SIRPα group. An unpaired Student’s t test was used to analyze the significance of the difference between two groups. Figure S5. Cell frozen lysate and CFS induce RAW264.7 polarization in vitro. A. Flow cytometric analysis results of one representative sample from each cell line. B. The ratio of M1 (F4/80+CD86+) subtype in the Cell frozen lysate and CFS groups of CT26, MC38 and 4T-1 cells detected by flow cytometry. An unpaired Student’s t test was used to analyze the significance of the difference between two groups. Figure S6. OH2 lysates induce primary macrophages polarization in vitro. A. Demonstration of the analysis of the polarization of macrophages by flow cytometry. B. The percentage of M1 (F4/80+CD86+) subtype and M [file 12916_2022_2574_MOESM3_ESM.zip › Additional file 3 Figure S17R3.tif]

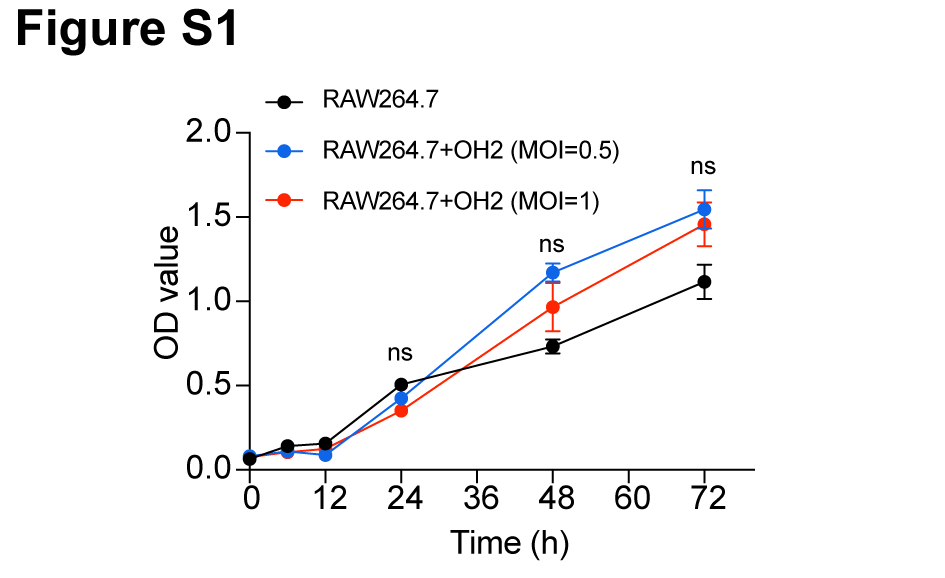

Supplement: Supplementary file 3 — Additional file 3: Figure S1. Cell proliferation assay results. Cell proliferation assay results of Raw264.7 cell lines treated with OH2 MOI=1 (red), OH2 MOI=0.5 (blue) and untreated (black) group by CCK8 assay in 72 hours. Figure S2. OH2 lysates induce RAW264.7 polarization and phagocytosis in vitro. A. Demonstration of the analysis of the phagocytosis by flow cytometry. B. Cell proliferation assay results of CT26, MC38 and 4T-1 cell lines treated with lysate (red), CFS (blue), Cell frozen lysate (purple) and untreated (black) group by CCK8 assay in 24 hours. **, p<0.01. Figure S3. The ratio of M1 (F4/80+CD86+) and M2 (F4/80+CD206+) macrophages in RAW264.7 without any treatment by flow cytometry. Figure S4. The ratio of M1 (F4/80+CD86+) and M2 (F4/80+CD206+) macrophages in RAW264.7 treated with lysate in the blocked SIRPα group and the non-blocked SIRPα group. A. Demonstration of the analysis of the polarization of macrophages by flow cytometry. B. Display of isotype control results for different antibodies. C. The ratio of M1 (F4/80+CD86+) and M2 (F4/80+CD206+) macrophages in the blocked SIRPα group and the non-blocked SIRPα group. D. The ratio of M1 (F4/80+CD86+) and M2 (F4/80+CD206+) macrophages in the blocked SIRPα group and the non-blocked SIRPα group. An unpaired Student’s t test was used to analyze the significance of the difference between two groups. Figure S5. Cell frozen lysate and CFS induce RAW264.7 polarization in vitro. A. Flow cytometric analysis results of one representative sample from each cell line. B. The ratio of M1 (F4/80+CD86+) subtype in the Cell frozen lysate and CFS groups of CT26, MC38 and 4T-1 cells detected by flow cytometry. An unpaired Student’s t test was used to analyze the significance of the difference between two groups. Figure S6. OH2 lysates induce primary macrophages polarization in vitro. A. Demonstration of the analysis of the polarization of macrophages by flow cytometry. B. The percentage of M1 (F4/80+CD86+) subtype and M [file 12916_2022_2574_MOESM3_ESM.zip › Additional file 3 Figure S1R3.tif]

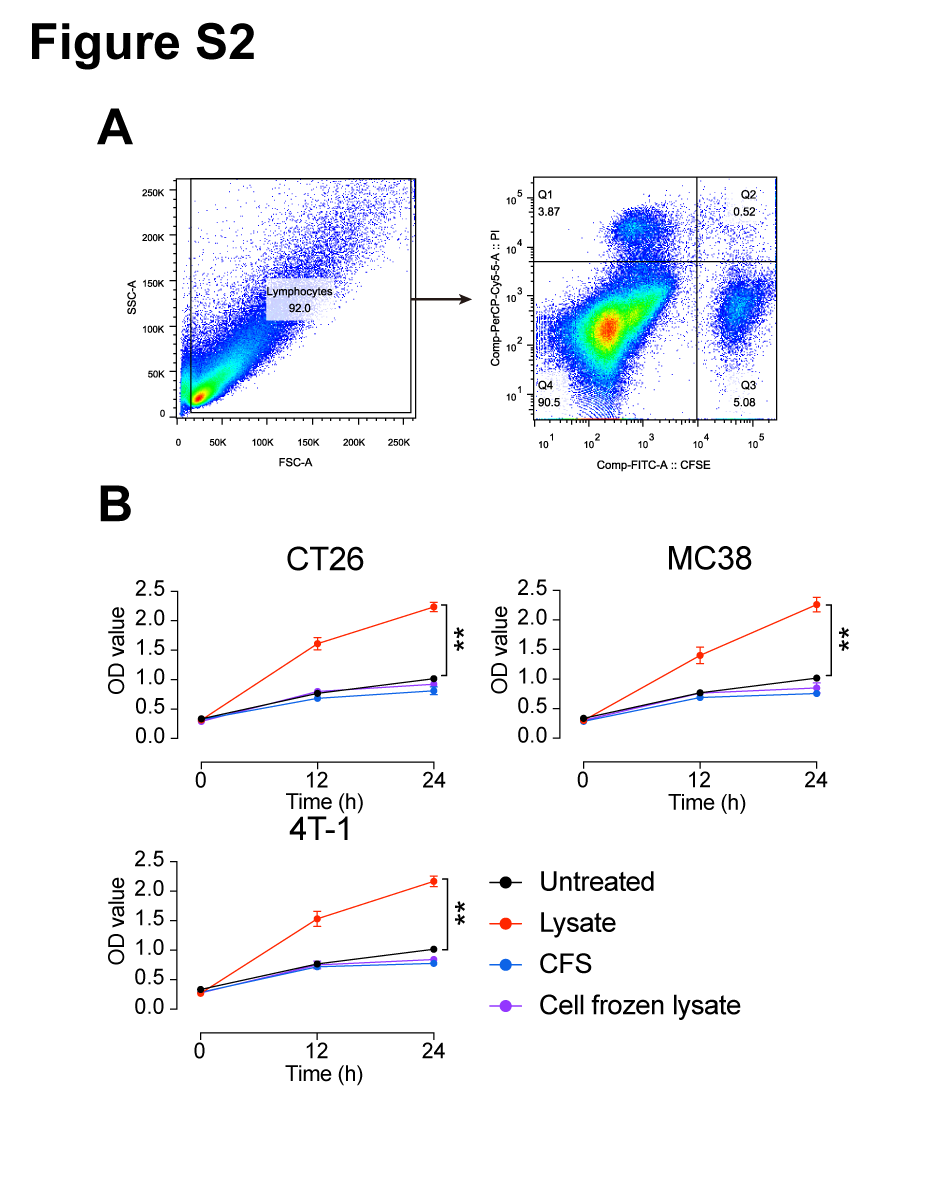

Supplement: Supplementary file 3 — Additional file 3: Figure S1. Cell proliferation assay results. Cell proliferation assay results of Raw264.7 cell lines treated with OH2 MOI=1 (red), OH2 MOI=0.5 (blue) and untreated (black) group by CCK8 assay in 72 hours. Figure S2. OH2 lysates induce RAW264.7 polarization and phagocytosis in vitro. A. Demonstration of the analysis of the phagocytosis by flow cytometry. B. Cell proliferation assay results of CT26, MC38 and 4T-1 cell lines treated with lysate (red), CFS (blue), Cell frozen lysate (purple) and untreated (black) group by CCK8 assay in 24 hours. **, p<0.01. Figure S3. The ratio of M1 (F4/80+CD86+) and M2 (F4/80+CD206+) macrophages in RAW264.7 without any treatment by flow cytometry. Figure S4. The ratio of M1 (F4/80+CD86+) and M2 (F4/80+CD206+) macrophages in RAW264.7 treated with lysate in the blocked SIRPα group and the non-blocked SIRPα group. A. Demonstration of the analysis of the polarization of macrophages by flow cytometry. B. Display of isotype control results for different antibodies. C. The ratio of M1 (F4/80+CD86+) and M2 (F4/80+CD206+) macrophages in the blocked SIRPα group and the non-blocked SIRPα group. D. The ratio of M1 (F4/80+CD86+) and M2 (F4/80+CD206+) macrophages in the blocked SIRPα group and the non-blocked SIRPα group. An unpaired Student’s t test was used to analyze the significance of the difference between two groups. Figure S5. Cell frozen lysate and CFS induce RAW264.7 polarization in vitro. A. Flow cytometric analysis results of one representative sample from each cell line. B. The ratio of M1 (F4/80+CD86+) subtype in the Cell frozen lysate and CFS groups of CT26, MC38 and 4T-1 cells detected by flow cytometry. An unpaired Student’s t test was used to analyze the significance of the difference between two groups. Figure S6. OH2 lysates induce primary macrophages polarization in vitro. A. Demonstration of the analysis of the polarization of macrophages by flow cytometry. B. The percentage of M1 (F4/80+CD86+) subtype and M [file 12916_2022_2574_MOESM3_ESM.zip › Additional file 3 Figure S2R3.tif]

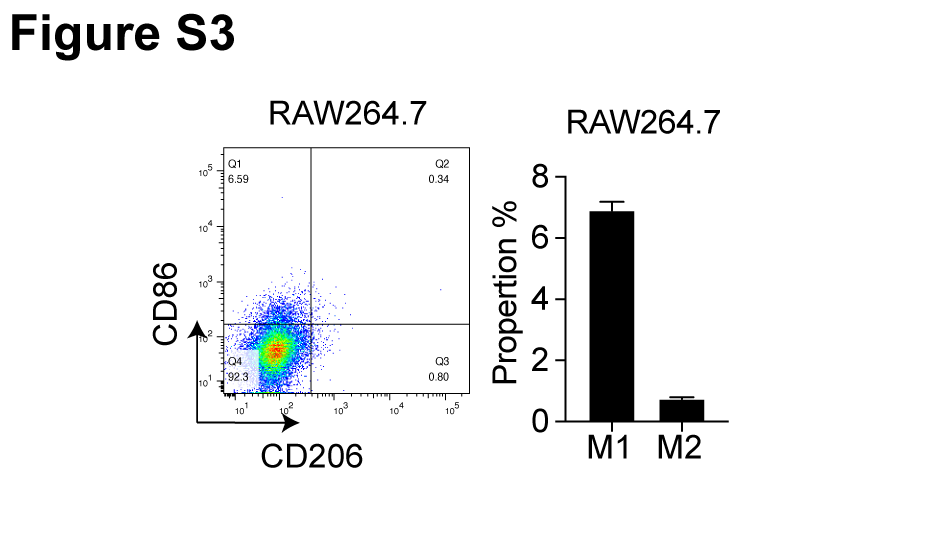

Supplement: Supplementary file 3 — Additional file 3: Figure S1. Cell proliferation assay results. Cell proliferation assay results of Raw264.7 cell lines treated with OH2 MOI=1 (red), OH2 MOI=0.5 (blue) and untreated (black) group by CCK8 assay in 72 hours. Figure S2. OH2 lysates induce RAW264.7 polarization and phagocytosis in vitro. A. Demonstration of the analysis of the phagocytosis by flow cytometry. B. Cell proliferation assay results of CT26, MC38 and 4T-1 cell lines treated with lysate (red), CFS (blue), Cell frozen lysate (purple) and untreated (black) group by CCK8 assay in 24 hours. **, p<0.01. Figure S3. The ratio of M1 (F4/80+CD86+) and M2 (F4/80+CD206+) macrophages in RAW264.7 without any treatment by flow cytometry. Figure S4. The ratio of M1 (F4/80+CD86+) and M2 (F4/80+CD206+) macrophages in RAW264.7 treated with lysate in the blocked SIRPα group and the non-blocked SIRPα group. A. Demonstration of the analysis of the polarization of macrophages by flow cytometry. B. Display of isotype control results for different antibodies. C. The ratio of M1 (F4/80+CD86+) and M2 (F4/80+CD206+) macrophages in the blocked SIRPα group and the non-blocked SIRPα group. D. The ratio of M1 (F4/80+CD86+) and M2 (F4/80+CD206+) macrophages in the blocked SIRPα group and the non-blocked SIRPα group. An unpaired Student’s t test was used to analyze the significance of the difference between two groups. Figure S5. Cell frozen lysate and CFS induce RAW264.7 polarization in vitro. A. Flow cytometric analysis results of one representative sample from each cell line. B. The ratio of M1 (F4/80+CD86+) subtype in the Cell frozen lysate and CFS groups of CT26, MC38 and 4T-1 cells detected by flow cytometry. An unpaired Student’s t test was used to analyze the significance of the difference between two groups. Figure S6. OH2 lysates induce primary macrophages polarization in vitro. A. Demonstration of the analysis of the polarization of macrophages by flow cytometry. B. The percentage of M1 (F4/80+CD86+) subtype and M [file 12916_2022_2574_MOESM3_ESM.zip › Additional file 3 Figure S3R3.tif]

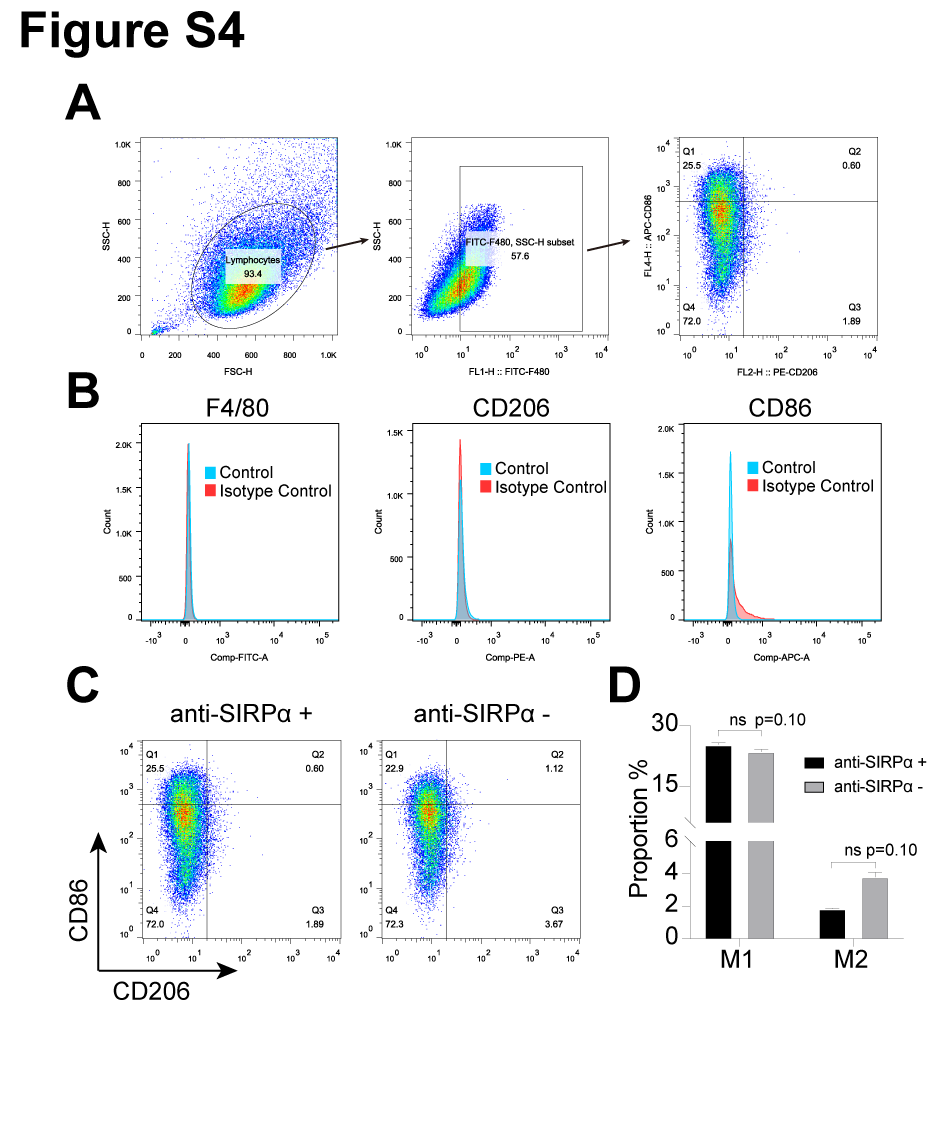

Supplement: Supplementary file 3 — Additional file 3: Figure S1. Cell proliferation assay results. Cell proliferation assay results of Raw264.7 cell lines treated with OH2 MOI=1 (red), OH2 MOI=0.5 (blue) and untreated (black) group by CCK8 assay in 72 hours. Figure S2. OH2 lysates induce RAW264.7 polarization and phagocytosis in vitro. A. Demonstration of the analysis of the phagocytosis by flow cytometry. B. Cell proliferation assay results of CT26, MC38 and 4T-1 cell lines treated with lysate (red), CFS (blue), Cell frozen lysate (purple) and untreated (black) group by CCK8 assay in 24 hours. **, p<0.01. Figure S3. The ratio of M1 (F4/80+CD86+) and M2 (F4/80+CD206+) macrophages in RAW264.7 without any treatment by flow cytometry. Figure S4. The ratio of M1 (F4/80+CD86+) and M2 (F4/80+CD206+) macrophages in RAW264.7 treated with lysate in the blocked SIRPα group and the non-blocked SIRPα group. A. Demonstration of the analysis of the polarization of macrophages by flow cytometry. B. Display of isotype control results for different antibodies. C. The ratio of M1 (F4/80+CD86+) and M2 (F4/80+CD206+) macrophages in the blocked SIRPα group and the non-blocked SIRPα group. D. The ratio of M1 (F4/80+CD86+) and M2 (F4/80+CD206+) macrophages in the blocked SIRPα group and the non-blocked SIRPα group. An unpaired Student’s t test was used to analyze the significance of the difference between two groups. Figure S5. Cell frozen lysate and CFS induce RAW264.7 polarization in vitro. A. Flow cytometric analysis results of one representative sample from each cell line. B. The ratio of M1 (F4/80+CD86+) subtype in the Cell frozen lysate and CFS groups of CT26, MC38 and 4T-1 cells detected by flow cytometry. An unpaired Student’s t test was used to analyze the significance of the difference between two groups. Figure S6. OH2 lysates induce primary macrophages polarization in vitro. A. Demonstration of the analysis of the polarization of macrophages by flow cytometry. B. The percentage of M1 (F4/80+CD86+) subtype and M [file 12916_2022_2574_MOESM3_ESM.zip › Additional file 3 Figure S4R3.tif]

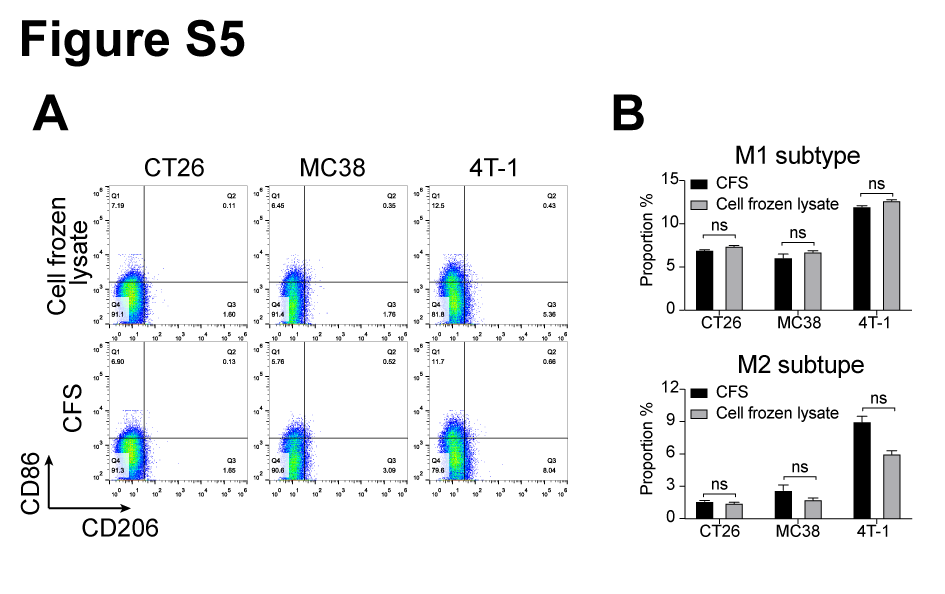

Supplement: Supplementary file 3 — Additional file 3: Figure S1. Cell proliferation assay results. Cell proliferation assay results of Raw264.7 cell lines treated with OH2 MOI=1 (red), OH2 MOI=0.5 (blue) and untreated (black) group by CCK8 assay in 72 hours. Figure S2. OH2 lysates induce RAW264.7 polarization and phagocytosis in vitro. A. Demonstration of the analysis of the phagocytosis by flow cytometry. B. Cell proliferation assay results of CT26, MC38 and 4T-1 cell lines treated with lysate (red), CFS (blue), Cell frozen lysate (purple) and untreated (black) group by CCK8 assay in 24 hours. **, p<0.01. Figure S3. The ratio of M1 (F4/80+CD86+) and M2 (F4/80+CD206+) macrophages in RAW264.7 without any treatment by flow cytometry. Figure S4. The ratio of M1 (F4/80+CD86+) and M2 (F4/80+CD206+) macrophages in RAW264.7 treated with lysate in the blocked SIRPα group and the non-blocked SIRPα group. A. Demonstration of the analysis of the polarization of macrophages by flow cytometry. B. Display of isotype control results for different antibodies. C. The ratio of M1 (F4/80+CD86+) and M2 (F4/80+CD206+) macrophages in the blocked SIRPα group and the non-blocked SIRPα group. D. The ratio of M1 (F4/80+CD86+) and M2 (F4/80+CD206+) macrophages in the blocked SIRPα group and the non-blocked SIRPα group. An unpaired Student’s t test was used to analyze the significance of the difference between two groups. Figure S5. Cell frozen lysate and CFS induce RAW264.7 polarization in vitro. A. Flow cytometric analysis results of one representative sample from each cell line. B. The ratio of M1 (F4/80+CD86+) subtype in the Cell frozen lysate and CFS groups of CT26, MC38 and 4T-1 cells detected by flow cytometry. An unpaired Student’s t test was used to analyze the significance of the difference between two groups. Figure S6. OH2 lysates induce primary macrophages polarization in vitro. A. Demonstration of the analysis of the polarization of macrophages by flow cytometry. B. The percentage of M1 (F4/80+CD86+) subtype and M [file 12916_2022_2574_MOESM3_ESM.zip › Additional file 3 Figure S5R3.tif]

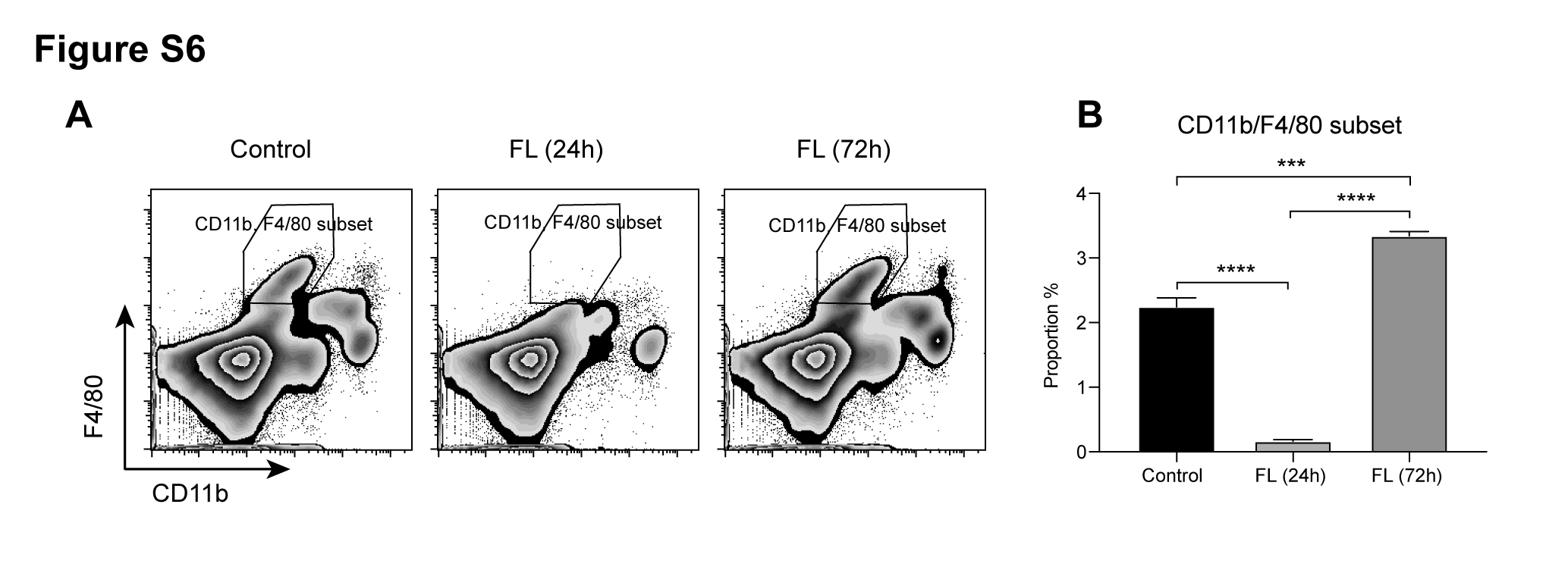

Supplement: Supplementary file 3 — Additional file 3: Figure S1. Cell proliferation assay results. Cell proliferation assay results of Raw264.7 cell lines treated with OH2 MOI=1 (red), OH2 MOI=0.5 (blue) and untreated (black) group by CCK8 assay in 72 hours. Figure S2. OH2 lysates induce RAW264.7 polarization and phagocytosis in vitro. A. Demonstration of the analysis of the phagocytosis by flow cytometry. B. Cell proliferation assay results of CT26, MC38 and 4T-1 cell lines treated with lysate (red), CFS (blue), Cell frozen lysate (purple) and untreated (black) group by CCK8 assay in 24 hours. **, p<0.01. Figure S3. The ratio of M1 (F4/80+CD86+) and M2 (F4/80+CD206+) macrophages in RAW264.7 without any treatment by flow cytometry. Figure S4. The ratio of M1 (F4/80+CD86+) and M2 (F4/80+CD206+) macrophages in RAW264.7 treated with lysate in the blocked SIRPα group and the non-blocked SIRPα group. A. Demonstration of the analysis of the polarization of macrophages by flow cytometry. B. Display of isotype control results for different antibodies. C. The ratio of M1 (F4/80+CD86+) and M2 (F4/80+CD206+) macrophages in the blocked SIRPα group and the non-blocked SIRPα group. D. The ratio of M1 (F4/80+CD86+) and M2 (F4/80+CD206+) macrophages in the blocked SIRPα group and the non-blocked SIRPα group. An unpaired Student’s t test was used to analyze the significance of the difference between two groups. Figure S5. Cell frozen lysate and CFS induce RAW264.7 polarization in vitro. A. Flow cytometric analysis results of one representative sample from each cell line. B. The ratio of M1 (F4/80+CD86+) subtype in the Cell frozen lysate and CFS groups of CT26, MC38 and 4T-1 cells detected by flow cytometry. An unpaired Student’s t test was used to analyze the significance of the difference between two groups. Figure S6. OH2 lysates induce primary macrophages polarization in vitro. A. Demonstration of the analysis of the polarization of macrophages by flow cytometry. B. The percentage of M1 (F4/80+CD86+) subtype and M [file 12916_2022_2574_MOESM3_ESM.zip › Additional file 3 Figure S6R3.tif]

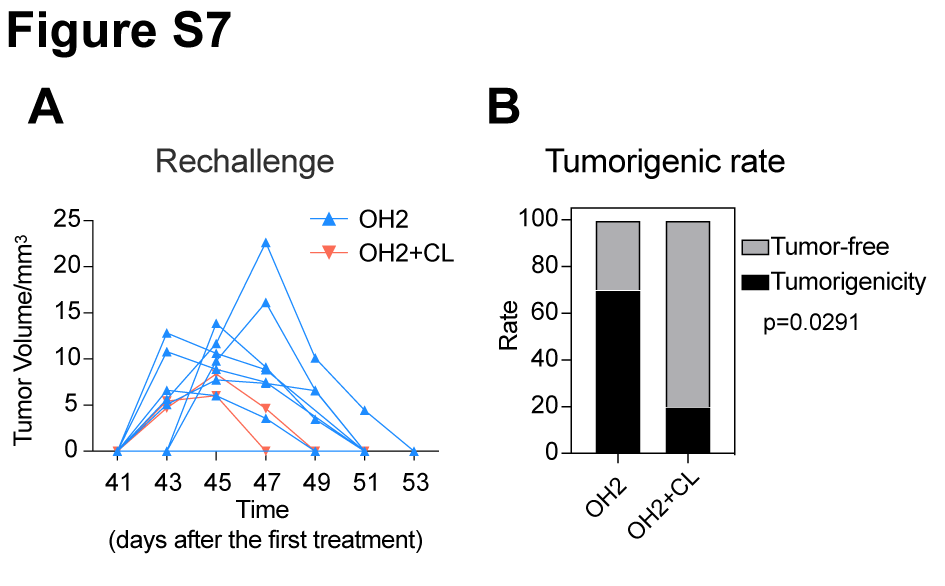

Supplement: Supplementary file 3 — Additional file 3: Figure S1. Cell proliferation assay results. Cell proliferation assay results of Raw264.7 cell lines treated with OH2 MOI=1 (red), OH2 MOI=0.5 (blue) and untreated (black) group by CCK8 assay in 72 hours. Figure S2. OH2 lysates induce RAW264.7 polarization and phagocytosis in vitro. A. Demonstration of the analysis of the phagocytosis by flow cytometry. B. Cell proliferation assay results of CT26, MC38 and 4T-1 cell lines treated with lysate (red), CFS (blue), Cell frozen lysate (purple) and untreated (black) group by CCK8 assay in 24 hours. **, p<0.01. Figure S3. The ratio of M1 (F4/80+CD86+) and M2 (F4/80+CD206+) macrophages in RAW264.7 without any treatment by flow cytometry. Figure S4. The ratio of M1 (F4/80+CD86+) and M2 (F4/80+CD206+) macrophages in RAW264.7 treated with lysate in the blocked SIRPα group and the non-blocked SIRPα group. A. Demonstration of the analysis of the polarization of macrophages by flow cytometry. B. Display of isotype control results for different antibodies. C. The ratio of M1 (F4/80+CD86+) and M2 (F4/80+CD206+) macrophages in the blocked SIRPα group and the non-blocked SIRPα group. D. The ratio of M1 (F4/80+CD86+) and M2 (F4/80+CD206+) macrophages in the blocked SIRPα group and the non-blocked SIRPα group. An unpaired Student’s t test was used to analyze the significance of the difference between two groups. Figure S5. Cell frozen lysate and CFS induce RAW264.7 polarization in vitro. A. Flow cytometric analysis results of one representative sample from each cell line. B. The ratio of M1 (F4/80+CD86+) subtype in the Cell frozen lysate and CFS groups of CT26, MC38 and 4T-1 cells detected by flow cytometry. An unpaired Student’s t test was used to analyze the significance of the difference between two groups. Figure S6. OH2 lysates induce primary macrophages polarization in vitro. A. Demonstration of the analysis of the polarization of macrophages by flow cytometry. B. The percentage of M1 (F4/80+CD86+) subtype and M [file 12916_2022_2574_MOESM3_ESM.zip › Additional file 3 Figure S7R3.tif]

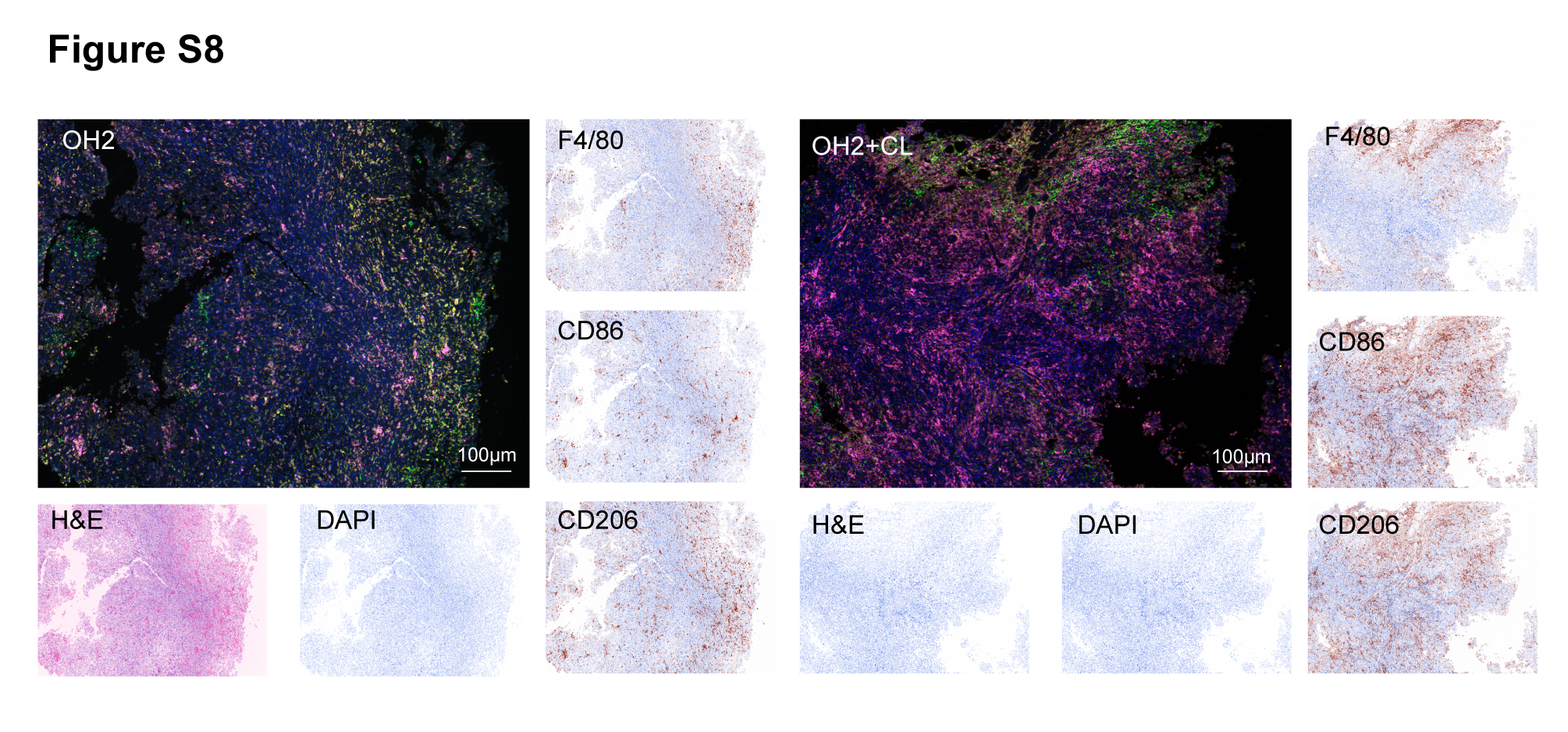

Supplement: Supplementary file 3 — Additional file 3: Figure S1. Cell proliferation assay results. Cell proliferation assay results of Raw264.7 cell lines treated with OH2 MOI=1 (red), OH2 MOI=0.5 (blue) and untreated (black) group by CCK8 assay in 72 hours. Figure S2. OH2 lysates induce RAW264.7 polarization and phagocytosis in vitro. A. Demonstration of the analysis of the phagocytosis by flow cytometry. B. Cell proliferation assay results of CT26, MC38 and 4T-1 cell lines treated with lysate (red), CFS (blue), Cell frozen lysate (purple) and untreated (black) group by CCK8 assay in 24 hours. **, p<0.01. Figure S3. The ratio of M1 (F4/80+CD86+) and M2 (F4/80+CD206+) macrophages in RAW264.7 without any treatment by flow cytometry. Figure S4. The ratio of M1 (F4/80+CD86+) and M2 (F4/80+CD206+) macrophages in RAW264.7 treated with lysate in the blocked SIRPα group and the non-blocked SIRPα group. A. Demonstration of the analysis of the polarization of macrophages by flow cytometry. B. Display of isotype control results for different antibodies. C. The ratio of M1 (F4/80+CD86+) and M2 (F4/80+CD206+) macrophages in the blocked SIRPα group and the non-blocked SIRPα group. D. The ratio of M1 (F4/80+CD86+) and M2 (F4/80+CD206+) macrophages in the blocked SIRPα group and the non-blocked SIRPα group. An unpaired Student’s t test was used to analyze the significance of the difference between two groups. Figure S5. Cell frozen lysate and CFS induce RAW264.7 polarization in vitro. A. Flow cytometric analysis results of one representative sample from each cell line. B. The ratio of M1 (F4/80+CD86+) subtype in the Cell frozen lysate and CFS groups of CT26, MC38 and 4T-1 cells detected by flow cytometry. An unpaired Student’s t test was used to analyze the significance of the difference between two groups. Figure S6. OH2 lysates induce primary macrophages polarization in vitro. A. Demonstration of the analysis of the polarization of macrophages by flow cytometry. B. The percentage of M1 (F4/80+CD86+) subtype and M [file 12916_2022_2574_MOESM3_ESM.zip › Additional file 3 Figure S8R3.tif]

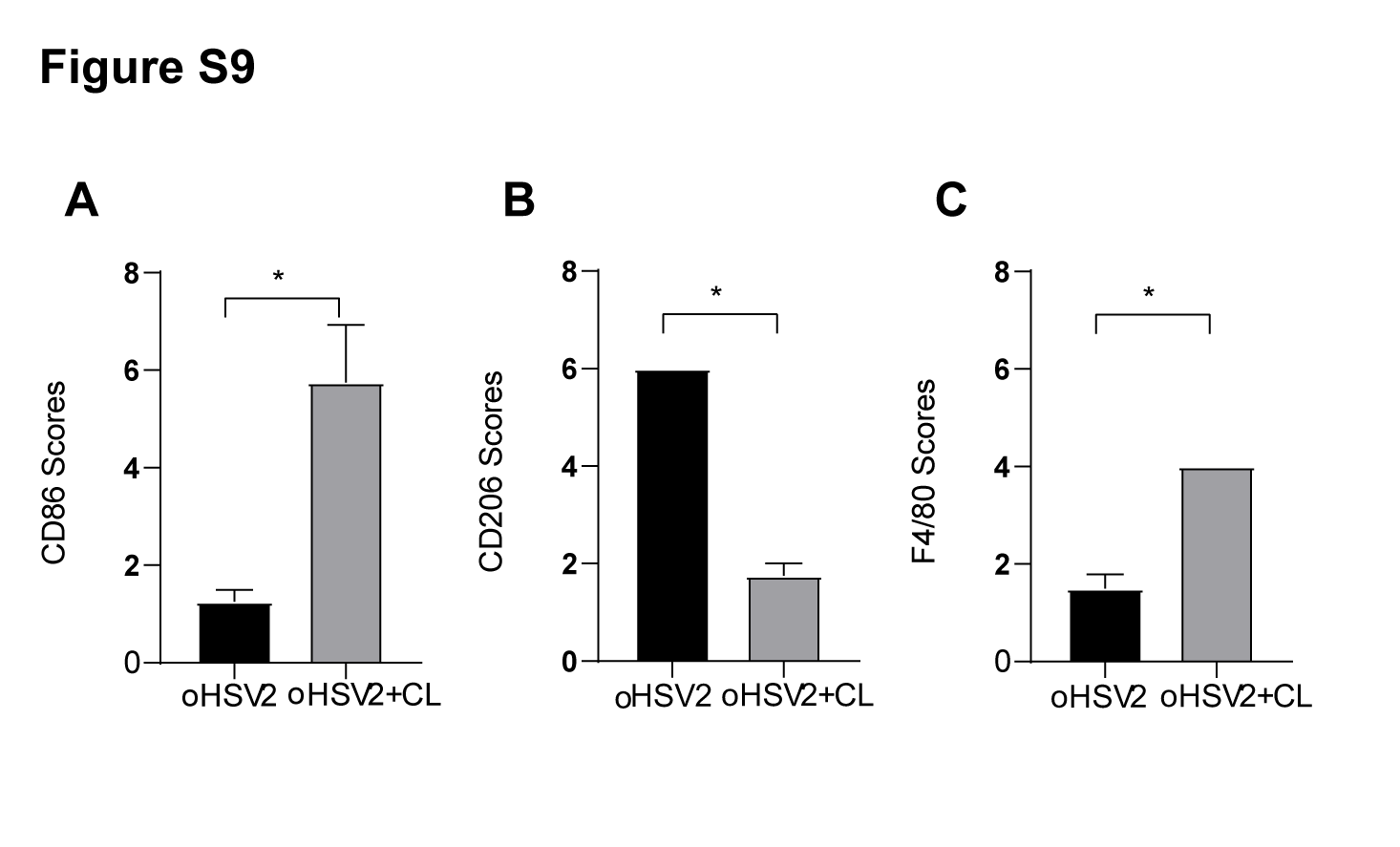

Supplement: Supplementary file 3 — Additional file 3: Figure S1. Cell proliferation assay results. Cell proliferation assay results of Raw264.7 cell lines treated with OH2 MOI=1 (red), OH2 MOI=0.5 (blue) and untreated (black) group by CCK8 assay in 72 hours. Figure S2. OH2 lysates induce RAW264.7 polarization and phagocytosis in vitro. A. Demonstration of the analysis of the phagocytosis by flow cytometry. B. Cell proliferation assay results of CT26, MC38 and 4T-1 cell lines treated with lysate (red), CFS (blue), Cell frozen lysate (purple) and untreated (black) group by CCK8 assay in 24 hours. **, p<0.01. Figure S3. The ratio of M1 (F4/80+CD86+) and M2 (F4/80+CD206+) macrophages in RAW264.7 without any treatment by flow cytometry. Figure S4. The ratio of M1 (F4/80+CD86+) and M2 (F4/80+CD206+) macrophages in RAW264.7 treated with lysate in the blocked SIRPα group and the non-blocked SIRPα group. A. Demonstration of the analysis of the polarization of macrophages by flow cytometry. B. Display of isotype control results for different antibodies. C. The ratio of M1 (F4/80+CD86+) and M2 (F4/80+CD206+) macrophages in the blocked SIRPα group and the non-blocked SIRPα group. D. The ratio of M1 (F4/80+CD86+) and M2 (F4/80+CD206+) macrophages in the blocked SIRPα group and the non-blocked SIRPα group. An unpaired Student’s t test was used to analyze the significance of the difference between two groups. Figure S5. Cell frozen lysate and CFS induce RAW264.7 polarization in vitro. A. Flow cytometric analysis results of one representative sample from each cell line. B. The ratio of M1 (F4/80+CD86+) subtype in the Cell frozen lysate and CFS groups of CT26, MC38 and 4T-1 cells detected by flow cytometry. An unpaired Student’s t test was used to analyze the significance of the difference between two groups. Figure S6. OH2 lysates induce primary macrophages polarization in vitro. A. Demonstration of the analysis of the polarization of macrophages by flow cytometry. B. The percentage of M1 (F4/80+CD86+) subtype and M [file 12916_2022_2574_MOESM3_ESM.zip › Additional file 3 Figure S9R3.tif]
