# Supplementary material for: SIRPα antibody combined with oncolytic virus OH2 protects against tumours by activating innate immunity and reprogramming the tumour immune microenvironment
Source: BMC Med. 2022 Oct 31;20:376. doi: 10.1186/s12916-022-02574-z (PMC9620659; doi:10.1186/s12916-022-02574-z)
Supplement: Supplementary file 4 — Additional file 4. Supplementary methods. [file 12916_2022_2574_MOESM4_ESM.docx]

**Supplementary methods**

**Raw264.7 cells viability with OH2 treatment**

The RAW264.7 cells in the logarithmic growth phase were spread on a 96-well plate at 1000 cells/well, and cultured for more than 6 hours. After the cells adhered, added the lysate or OH2 (MOI=0.5 and 1) to the cells respectively, and performed Cell Counting Kit-8 (CCK8) detection at the corresponding time points (0 hours, 6 hours, 12 hours, 24 hours, 48 hours, 72 hours) (Dojindo, Kumamoto, Japan). Before detection, add 100ul detection working solution (CCK8 reagent: RPMI1640 medium = 1:10) to each well, and incubate for 1 hour in a 37°C, 5% CO2 incubator in the dark. Finally, a microplate reader (Bio-Rad, Japan) was used to detect the absorbance of the cells at a wavelength of 450nm.

**Determination of the clearance efficiency of CL by flow cytometry**

Female BALB/c mice aged 6-8 weeks were divided into three groups with 3 mice in each group. Group A was injected with CL100 μl/mouse on the first day, group B was given CL100 μl/mouse on the third day, and group C was given control liposome 100 μl/mouse on the third day. The proportion of CD11b+F4/80+ cells in the spleen of mice was detected by flow cytometry on the 4th day. Mice were sacrificed by cervical dislocation., the spleen was removed, and the spleen was ground with a sterile grinding rod. Then, the spleen cells were filtered through a 40 μm filter into a 15 ml centrifuge tube, centrifuged at 1500 rpm/min for 5 min at room temperature. The erythrocyte lysate was added to lyse the erythrocytes at room temperature for 8 min and then centrifuged at 1500 rpm/min for 5 min at room temperature to discard the erythrocyte lysate. The cells were washed twice with PBS to remove the residual erythrocyte lysate, and the cells were resuspended in 100 μl of PBS and incubated with the antibodies (FITC anti-mouse F4/80 (clone: FJK-16s, Invitrogen, Waltham, Massachusetts); APC anti-mouse CD11b (clone: M1/70, Biolegend, San Diego, CA)) at room temperature for 30 min. After the antibody incubation, the cells were washed twice with PBS, and the ratio of CD11b+F4/80+ cells was detected by flow cytometry.

**Isolation of mouse spleen primary macrophage**

Six-week-old female Balb/c mice (n=3) were sacrificed, and spleens were collected. The spleens were grinded in cold PBS and then filtered through a 70-μm cell strainer. F4/80+ macrophages were isolated using an anti-F4/80 MicroBeads (Miltenyi Biotec, Germany) according to the manufacturer’s instructions.

**Flow cytometry to detect the polarization direction of primary macrophages**

Primary F4/80+ macrophages cells were spread to a 12-well plate at 5×10^5^ cells/well. After at least 6 hours the lysate, CFS, cell frozen lysate, and control were added separately. After 24 hours of treatment, cells were collected and stained with FITC anti-mouse F4/80 (clone: FJK-16s, Invitrogen, Waltham, Massachusetts), APC anti-mouse CD86 (clone: GL-1, Biolegend, San Diego, CA) and PE anti-mouse CD206 (clone: MR6F3, Invitrogen, Waltham, Massachusetts) according to the protocol of the antibodies (M1 macrophage: F4/80+CD86+, M2 macrophage: F4/80+CD206+) and subjected to flow cytometry (LSR II, BD).

**In vivo induced CTL and CTL assay**

Seven days after final treatment of tumor-bearing CT-26 mice (n=3 per group), spleens were harvested. Splenic lymphocytes were isolated using lymphocyte separation medium (Dakewe Biotech, China) at room temperature and washed twice with PBS. CT26 cells were labeled with 0.5 mmol/L CFSE for 8 min at 37℃. After terminated, the cells were collected and adjusted to 4×10^5^ cells/mL. Then, lymphocyte effector cells and target cells were mixed at E:T ratios of 100:1, 50:1, and 25:1. After incubation at 37℃ and 5% CO_2_ for 4 h, cells were harvested and labeled with 1 mg/mL PI for 5 min at room temperature, subjected to flow cytometry (LSR II, BD).

For cytokine analysis, 7 days after final treatment of tumor-bearing CT-26 mice (n=5 per group), splenic lymphocytes were isolated using lymphocyte separation medium (Dakewe Biotech, China) at room temperature and washed twice with PBS. Splenic lymphocytes were adjusted to 1×10^7^ cells/mL and co-cultured with 1×10^6^ CT-26 cells in RPMI with 10% FCS in a 6-well plate for 48 hours. The Granzyme B, TNF-α and IFN-γ in supernatant were analyzed by ELISA (Jingmei Biotechnology, China).
